# Comparative transcriptomic profiling of hydrogen peroxide signaling networks in zebrafish and human keratinocytes: Implications toward conservation, migration and wound healing

**DOI:** 10.1038/srep20328

**Published:** 2016-02-05

**Authors:** Thomas S. Lisse, Benjamin L. King, Sandra Rieger

**Affiliations:** 1 Davis Center for Regenerative Biology and Medicine, MDI Biological Laboratory, Kathryn W. Davis Building 227, Old Bar Harbor Road, Salisbury Cove, Maine 04672, U.S.A.; 2 The Jackson Laboratory, Bar Harbor, Maine 04609, U.S.A.

## Abstract

Skin wounds need to be repaired rapidly after injury to restore proper skin barrier function. Hydrogen peroxide (H_2_O_2_) is a conserved signaling factor that has been shown to promote a variety of skin wound repair processes, including immune cell migration, angiogenesis and sensory axon repair. Despite growing research on H_2_O_2_ functions in wound repair, the downstream signaling pathways activated by this reactive oxygen species in the context of injury remain largely unknown. The goal of this study was to provide a comprehensive analysis of gene expression changes in the epidermis upon exposure to H_2_O_2_ concentrations known to promote wound repair. Comparative transcriptome analysis using RNA-seq data from larval zebrafish and previously reported microarray data from a human epidermal keratinocyte line shows that H_2_O_2_ activates conserved cell migration, adhesion, cytoprotective and anti-apoptotic programs in both zebrafish and human keratinocytes. Further assessment of expression characteristics and signaling pathways revealed the activation of three major H_2_O_2_–dependent pathways, EGF, FOXO1, and IKKα. This study expands on our current understanding of the clinical potential of low-level H_2_O_2_ for the promotion of epidermal wound repair and provides potential candidates in the treatment of wound healing deficits.

The skin forms a barrier that prevents water loss and protects against the external environment^1^. This barrier is compromised upon injury, whereby a dynamic multi-step process is stimulated involving modulation of complex gene regulatory networks and cellular interactions by local trophic and endocrine factors^2^. Wound repair consists of three major overlapping, yet distinct phases – inflammation, tissue formation and remodeling – that need to be orchestrated to achieve proper tissue repair and regain protection from opportunistic infections^3^. During the early stages of repair, re-epithelialization or resurfacing of wounds occurs to restore the epidermal barrier^4^,^5^. This step involves migration and proliferation of keratinocytes from the surrounding epidermis, which undergo pronounced genomic and molecular changes. For example, as keratinocytes begin to migrate in response to acute injury, basal keratinocytes near the excisional wound margin in the migrating “tongue” express a distinct pattern of keratins reflecting their activation state toward wound closure^6^.

A number of soluble mediators, including epidermal and platelet-derived growth factors, are known to influence the re-epithelialization process by making the extracellular and intracellular environment more permissible to repair^3^,^7^,^8^,^9^. Other factors include the small reactive oxygen species (ROS) hydrogen peroxide (H_2_O_2_)^10^, but its intrinsic mechanisms of action at both the genomic and molecular levels have remained largely unexplored. All aerobic organisms routinely experience physiological, stress and environmental conditions that provoke the accumulation and decomposition of ROS. H_2_O_2_ was initially recognized as a toxic oxygen derivative and byproduct of normal metabolism, capable of assaulting a range of vital cellular structures and biomolecules^11^. However in time, it became clear that H_2_O_2_ can also behave as a signaling molecule to regulate numerous conserved biological functions from proliferation, senescence to programmed cell death. For example, low concentrations of H_2_O_2_ can increase DNA synthesis within resting rat vascular smooth muscle cells through the expression of proto-oncogenes, *c-fos* and *c-myc*^12^. H_2_O_2_ signal transduction is evolutionarily conserved and is relayed through cells via receptors, protein kinases, structural components and downstream transcription factor-dependent mechanisms^13^,^14^. A classic example is the cysteine oxidation of the c-jun N-terminal kinase (JNK) that activates the AP-1 transcription factor upon elevated levels of H_2_O_2_, which modulates transcriptional responses across species with distinct biological outcomes^15^. Importantly, target cysteine oxidation can be assessed by detection of sulfenic acid intermediates, as was recently shown for epidermal growth factor receptor (EGFR) after H_2_O_2_ treatment^16^. Moreover across non-vertebrate species, plants have evolved the multifunctional use of lower concentrations of ROS to regulate growth and developmental processes, such as cell elongation^17^ and adaptations in response to environmental conditions^18^, and the hypersensitive response to wounds^19^ that involves activation cascades of multiple kinases and transcription factors.

Zebrafish has been widely used as a vertebrate model to study how the organism utilizes H_2_O_2_ to alert and mobilize cells when a tissue has been injured and needs repair. Within the complex cutaneous wound milieu, a number of immune cell types are recruited to the wound area by H_2_O_2_. For example, in zebrafish the Src family kinase Lyn is a known redox sensor in neutrophils that regulates neutrophil recruitment toward the wound^20^. It has been proposed that this process is regulated by a tissue-scale gradient of H_2_O_2_ emanating from the wound^21^. H_2_O_2_ gradients were recently found to be crucial for neutrophil reverse migration in wounded zebrafish^22^. In addition to its functions in immune cell migration, low levels of H_2_O_2_ (0.01%) are known to promote cutaneous sensory axon repair in injured zebrafish^23^. The overall beneficial effects of H_2_O_2_ are conserved across species, as recent studies have shown that “low” (0.5%) but not “high” (3%) H_2_O_2_, which is typically applied to human wounds to avoid infections, accelerates wound closure and angiogenesis in mice^24^. Yet, significant attenuation of H_2_O_2_ through overexpression of the antioxidant catalase delays wound healing in mice^25^, suggesting that narrow concentrations of H_2_O_2_ are key to rapid wound repair. *In vitro* studies using “scratched” keratinocytes^26^, corneal epithelial cells^27^, and a keratinocyte-fibroblast pseudo-wound healing co-culture^28^ system further suggest that low concentrations of exogenous H_2_O_2_ significantly accelerate keratinocyte migration during scratch wound repair in higher organisms.

The motogenic mechanisms of H_2_O_2_-induced keratinocyte migration are still largely unclear. Classically, keratinocyte migration has been studied from the perspective of growth factor-activated kinase signaling. For example, hepatocyte growth factor or EGF-stimulated human epidermal keratinocytes require extracellular signal-regulated kinase (ERK), but not JNK-AP-1, activation to stimulate cell molitlity^29^. Furthermore, these signaling processes which promote motility are highly dependent on specialized extracellular matrix (ECM)-driven factors^30^. In terms of H_2_O_2_, by using the human keratinocyte line HaCaT, Loo *et al.* demonstrated that H_2_O_2_ can activate downstream ERK1/2 phosphorylation cascades via EGFR activation, stimulating an increase in both proliferation and migration independent of the stress sensor p38 MAPK^26^. In part, H_2_O_2_ may also involve the indirect activation of the EGFR by targeting and blocking receptor-type protein-tyrosine phosphatases^31^. In addition, H_2_O_2_ was recently shown to directly phosphorylate and oxidize the EGFR catalytic site for its activation in epidermoid carcinoma cells^16^, linking the EGFR as a major upstream oxidation target for cellular responses.

Under basal conditions, ROS/H_2_O_2_ are generated largely in the mitochondrial electron transport chain where molecular oxygen is converted into free radicals and subsequently degraded to water and oxygen. In the cytoplasm, H_2_O_2_ production is regulated by NADPH oxidases (NOXs) that are located at the plasma membrane. NOX enzymes transfer electrons derived from NADPH to molecular oxygen to generate superoxide radicals and ultimately H_2_O_2_. From a health perspective, altered ROS generation and catabolism lead to pathological conditions such as atherosclerosis, Parkinson’s disease, Alzheimer’s disease and aging^32^,^33^,^34^,^35^. NOX activation after injury is essential to promote wound repair. Overall, the precise regulation of H_2_O_2_ levels within cells is essential for various cellular functions and wound repair, whereas uncontrolled production leads to disease. Here we provide a comprehensive evaluation of the genome-wide effects of low-level H_2_O_2_ in epithelial keratinocytes by comparing gene signatures in zebrafish using RNA-seq with a previously published human keratinocyte cell line that had been analyzed via oligonucleotide microarrays^36^. Our findings elaborate on the complexity of the transcriptional response to H_2_O_2_ within the cutaneous environment among vertebrate species.

## Results

### Comprehensive whole transcriptome RNA-seq analysis of larval zebrafish in response to low-level H_2_O_2_ stimulation

**Figure 1:**
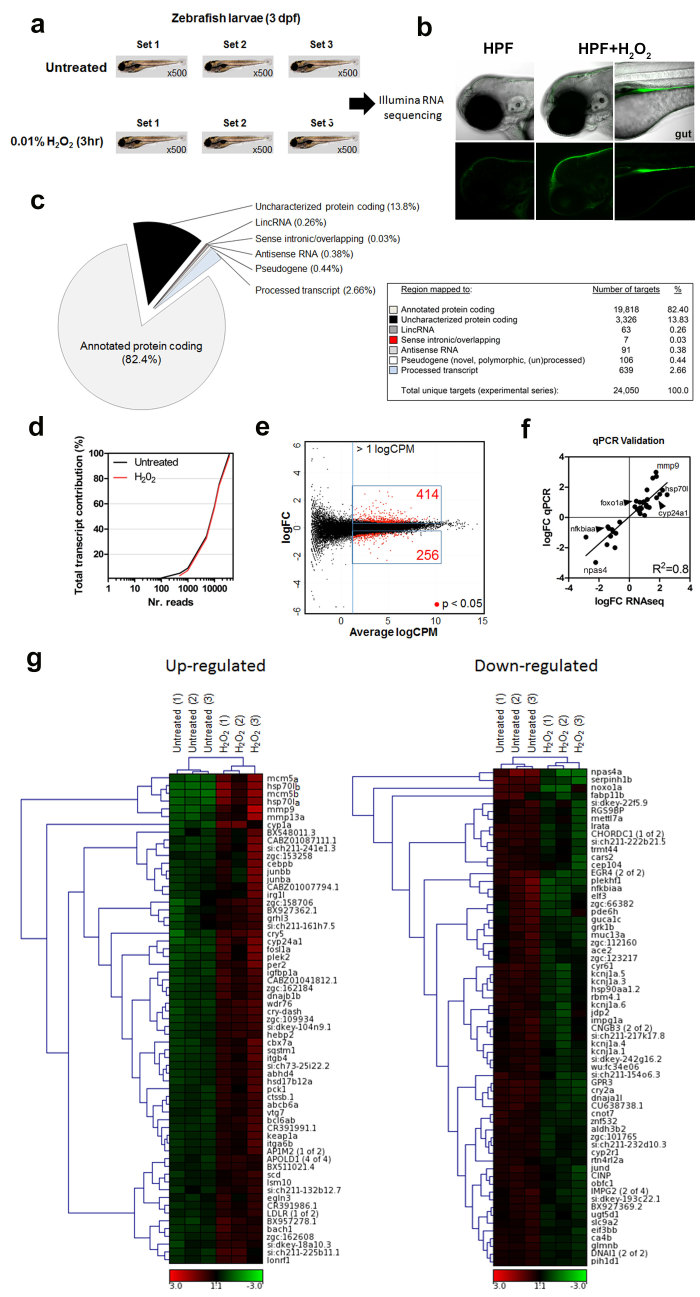
Whole transcriptome RNA-seq profile of larval zebrafish in response to low H_2_O_2_ treatment. (**a**) Pools of ~500 larvae/set of 4 day-post-fertilized (dpf) zebrafish larvae were treated with 0.01% (3 mM) H_2_O_2_ for three hours and total RNA was subsequently collected followed by pair-end next generation RNA sequencing (n = 3 biological replicates). (**b**) H_2_O_2_ sensor (HPF) either alone or with H_2_O_2_ treatment shows that H_2_O_2_ is mostly retained in the skin epithelium (n = 5 fish). (**c**) Distribution of mapped reads in the zebrafish transcriptome. RNA-seq data sent to NCBI (GEO: GSE75728). (**d**) Transcript complexity between untreated and H_2_O_2_-treated larval zebrafish samples. Based on read CPM (counts per million), the left-most value on the X-axis represents the most highly expressed transcripts, which is incrementally summed with each successively lower expressed transcript (rightward). The y-axis (% contribution of the total transcripts) was calculated using: [CPM/sum of all CPM] x 100%. (**e**) RNA-seq data was normalized with the read CPM method of the number of mapped reads on gene exons. Transcript expression data transformed on M (log ratio of fold change) and A (mean average) scale. Boxed blue regions represent statistically significant transcripts (p < 0.05) returned by the test for differential expression. The MA-plot shows the log_2_ fold changes from the treatment over the mean of normalized counts, i.e. the average of counts normalized by size factor. Cutoff set to >1 log_2_ CPM averaged over all samples, and below the cutoff there is no real inferential power. Note: Statistical significance drops below the threshold. (**f**) Quantitative PCR validation of RNA-seq results of a sub-set of candidate targets. Full data set is presented in Table S7 (n = 3 biological replicates). (**g**) Heat map indicates unsupervised hierarchical clustering of the top (left) and bottom (right) most significantly enriched transcripts derived from the RNA-seq data after H_2_O_2_ treatment. Hierarchical clustering was performed between individual experiments and transcripts. The color key indicates the log_2_ CPM expression values. *Abbreviations: HPF (hydrogen peroxide fluorogenic probe or pentafluorobenzenesulfonyl-fluorescein), CPM: Counts per million, FC: Fold-change*.

Appraisal of whole transcriptomic changes upon H_2_O_2_ treatment using next generation sequencing (NGS) approaches is appropriate to dissect genomic and molecular pathways at an ultra-sensitive level, and has yet to be applied for this purpose to date. To better understand the role of low H_2_O_2_ levels on signaling pathways, we performed genome-wide transcriptional analyses using RNA-seq to compare untreated wildtype 4 day post fertilization (dpf) zebrafish larvae in the absence of injury and upon treatment for 3hr with 3 mM (0.01%) H_2_O_2_ (Fig. 1). We previously demonstrated that incubation of larval zebrafish for up to 12 hours in 3 mM H_2_O_2_ promotes intra-epidermal sensory axon regeneration^23^, suggesting that this concentration of H_2_O_2_ is highly beneficial for tissue restoration in zebrafish larvae. We next assessed H_2_O_2_ diffusion properties with the H_2_O_2_-selective chemical sensor pentafluorobenzenesulfonyl-fluorescein (HPF) to determine potential tissues relevant for our gene expression analysis. While larvae treated with the sensor but not H_2_O_2_ exhibited no fluorescence, treatment with H_2_O_2_ led to selective fluorescence in the skin epithelium and gut, presumably due to ingestion (Fig. 1b). Therefore, differential gene expression induced by H_2_O_2_ should largely reflect changes in these tissues.

For RNA-seq, paired-end reads were aligned to the zebrafish transcriptome annotated by Ensembl (version 73)^37^ from the Zv9 zebrafish genome assembly^38^ using RSEM^39^ to generate read counts per million (CPM) for each transcript and gene. 11,736 genes out of the 35,786 genes annotated were not expressed as they had zero CPM for all six samples analyzed. Expressed genes consisted of annotated protein coding genes (~82.4%), uncharacterized protein coding genes (13.8%), processed transcripts (2.6%), pseudogenes (0.4%), antisense RNAs (0.4%), long intergenic non-coding RNAs (lincRNAs; 0.3%), and sense intronic (0.03%) elements (Fig. 1c). In general, processed transcripts have no known open reading frame with unique structures, lincRNAs are longer than 200 nucleotides, antisense RNAs intersect any exon of a protein coding locus on the opposite strand, and sense intronic loci reside within coding gene introns but do not intersect any exons on the same strand.

To determine any potential aberrant transcriptional impact of H_2_O_2_ treatment on zebrafish larvae, we evaluated the complexity of transcripts under the experimental conditions (Fig. 1d). By calculating the contribution of cumulative reads to the total transcript (i.e. % contribution to the total transcript) between untreated and H_2_O_2_-treated samples, we observed a complete overlap in the analysis, suggesting that changes in the transcriptome are biologically-relevant and specific to the treatment. Using log_2_-transformed CPM values determined for each sample by RSEM, differential expression was determined using R/edgeR^40^. Gene expression data was transformed and visualized on a M (difference between log intensities)/A (average mean) scale using CPM metrics (Fig. 1e). Using statistical and expression criteria (p < 0.05 and log_2_CPM > 1 respectively), 670 transcripts were identified as being differentially regulated when comparing H_2_O_2_ vs. untreated samples, of which 414 were significantly up and 256 were significantly downregulated (Worksheet 1). Among the differentially regulated transcripts, 56 were considered “uncharacterized” displaying some homology to mammalian counterparts, which require future validation (Table S5). These could also be transcripts aligned to genomic regions without transcript information or have no associated gene names. The 10 most H_2_O_2_-induced up and down regulated transcripts are reported in Table S1, and an expanded 40-most affected transcripts are presented as supplementary material (Table S6). Many of the statistically significant biologically-relevant transcripts (i.e. including the top/bottom-most transcripts↑hsp70l and ↓npas4) uncovered by RNA-seq were validated using conventional qPCR methods (Fig. 1f; Tables S1 and S7). These transcripts were later found to be part of regulatory networks associated with specialized and enriched biological roles (Section 5).

### Evaluation of the top-most up and down regulated genes after H_2_O_2_ treatment of larval zebrafish

The most elevated transcript after H_2_O_2_ treatment of zebrafish larvae was hsp70l (Tables S1 and S6; Worksheet 1), a heat shock protein involved in regulation of the cell cycle and cytoprotection after stress through protein folding mechanisms and inhibition of apoptosis^41^. Other major upregulated transcripts include mmp9 and mmp13a, known matrix metalloproteinases involved in embryonic development and tumor cell motility^42^. Moreover, Mmp13 has a known role in re-epithelialization in mice where it is specifically upregulated in the leading edge of migrating keratinocytes^43^. Also, cry5, a cryptochrome light-sensitive class of conserved flavoproteins that can act as transcriptional repressors within the circadian clockwork^44^, was significantly elevated after H_2_O_2_ treatment. Interestingly, overexpression of another cryptochrome (cry1) in colorectal tumors correlates with metastasis formation^45^. The molecular and biological link between increased *cry5* after H_2_O_2_ treatment is unclear, but may point to a light-induced H_2_O_2_ signaling role after skin wounding, possibly involving migration. Next, the ~60 most top and bottom statistically significant transcripts regulated by H_2_O_2_ were evaluated using hierarchical clustering to identify tendential patterns between the experiments and individual transcripts (Fig. 1g and Table S6). A gene cluster that showed the greatest difference between untreated and treated samples include *hsp70l, mmp9, mmp13* and *mcm5*. Of note, mcm5 (minichromosome maintenance complex component 5) is a chromatin-binding protein implicated in the initiation of DNA replication^46^, suggesting pro-mitotic effects of H_2_O_2_ treatment. Interestingly, although spanning several clusters, members of the mitochondrial cytochrome P450 superfamily of enzymes (i.e. *cyp1a* and *cyp24a1*) showed large expression differences between untreated and H_2_O_2_-treated samples. Cyp1a is involved in the metabolism of xenobiotic substrates, while Cyp24a1 initiates the catabolism of 1,25-dihydroxyvitamin D_3_ (1,25D_3_), the physiologically active form of vitamin D^47^, a well-known steroid hormone with anti-proliferative effects enriched in skin^48^,^49^,^50^,^51^. The latter suggests that the vitamin D system is suppressed during H_2_O_2_-induced molecular responses (see Discussion).

Amongst the most downregulated gene clusters upon H_2_O_2_ treatment were *npas4a* and *serpinh1b*. Npas4 (neuronal PAS domain protein 4) is a transcriptional activator that modulates cytoskeletal gene expression^52^ and is induced during ischemic tissue injury^53^, yet its role in the context of H_2_O_2_ signal transduction is unclear. Serpinh1 (serpin peptidase inhibitor, clade H, member 1; also called heat shock protein 47) is localized in the endoplasmic reticulum and acts as a molecular chaperone in the collagen biosynthetic pathway^54^, whereby a decrease in fibrosis is part of the cellular migratory phase during injury^2^. Thus, H_2_O_2_-depdendent downregulation of *serpinh1* may be involved in matrix integrity, composition and cell-matrix interactions important for cell migration and spreading.

**Figure 2:**
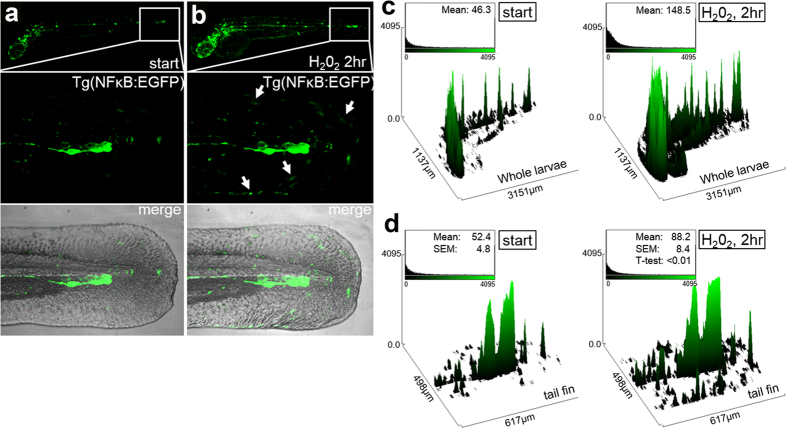
Transgenic NF-κB:EGFP zebrafish reveal peripheral NF-κB activation after H_2_O_2_ treatment Spatial patterns of increased NF-κB activation after H_2_O_2_ treatment of zebrafish larvae. (**a**) 3-dpf Tg(NF-κB:EGFP) reporter zebrafish larvae^59^ at the start of the experiment. (**b**) Tg(NF-κB:EGFP) zebrafish were imaged after 2hr post 0.01% H_2_O_2_ treatment. Both whole larvae (upper) and tail fins (below) were imaged, whereby GFP^+^ cells were partially localized to the periphery (arrows). (**c**) Quantitative analysis using relative mean fluorescence of the z-stack projected images using ImageJ (n = 2, 10 fish). Observation of increased number of GFP-labeled cells and overall fluorescence intensity in the whole larvae. (**d**) Higher-resolution analyses of the tail fin revealed a peripheral tissue spatial pattern of increase NF-κB activated cells after H_2_O_2_ treatment. n = 3 independent experiments per condition.

NF-κB activation can occur through multiple mechanisms and has been shown to require the generation of reactive oxygen intermediates (ROI)^55^. Previous findings have shown that H_2_O_2_ can induce NF-κB activation via Syk-mediated tyrosine phosphorylation of IκBα, an inhibitor of NF-κB, in transformed myeloid cells lines^56^. It is also known that H_2_O_2_ prolongs NF-κB nuclear localization by suppressing its export through polyubiquitination of signaling intermediates^57^. *nfkbiaa* (nuclear factor of kappa light polypeptide gene enhancer in B-cells inhibitor, alpha [IκBα] a), an inhibitor of NF-κB signaling and homologue to human IκBα^58^, was found to be one of the most down-regulated transcripts in our study (Table S1). Also, *nfkbiaa* formed a top-ranking downregulated gene cluster along with *plekhf1* and *elf3* after H_2_O_2_ treatment (Fig. 1f). To determine if indeed the NF-κB signaling pathway was activated in zebrafish treated with H_2_O_2_, we utilized a transgenic NF-κB:EGFP reporter line^59^ (Fig. 2). After 2hr of H_2_O_2_ treatment, the Tg(NF-κB:EGFP) reporter line resulted in both an increase in the mean fluorescence and the number of GFP^+^ cells localized to the periphery of the tail fin (Fig. 2a–d). As the actual identity of the GFP^+^ cells is unclear, our enriched pathway analysis of the RNA-seq data suggests an increase in immune cell trafficking as reflected by changes in expression of 24 implicated genes (Table S2). Thus, H_2_O_2_ prolongs NF-κB activity in larval zebrafish, in part, by suppressing negative transcriptional regulatory mechanisms. Our findings point to a potential alternative transcriptional regulatory role of H_2_O_2_, apart from post-translational events, in the control of NF-κB signaling during inflammatory responses.

### Identification of enriched H_2_O_2_-induced transcriptional and biological pathways in zebrafish larvae

**Figure 3:**
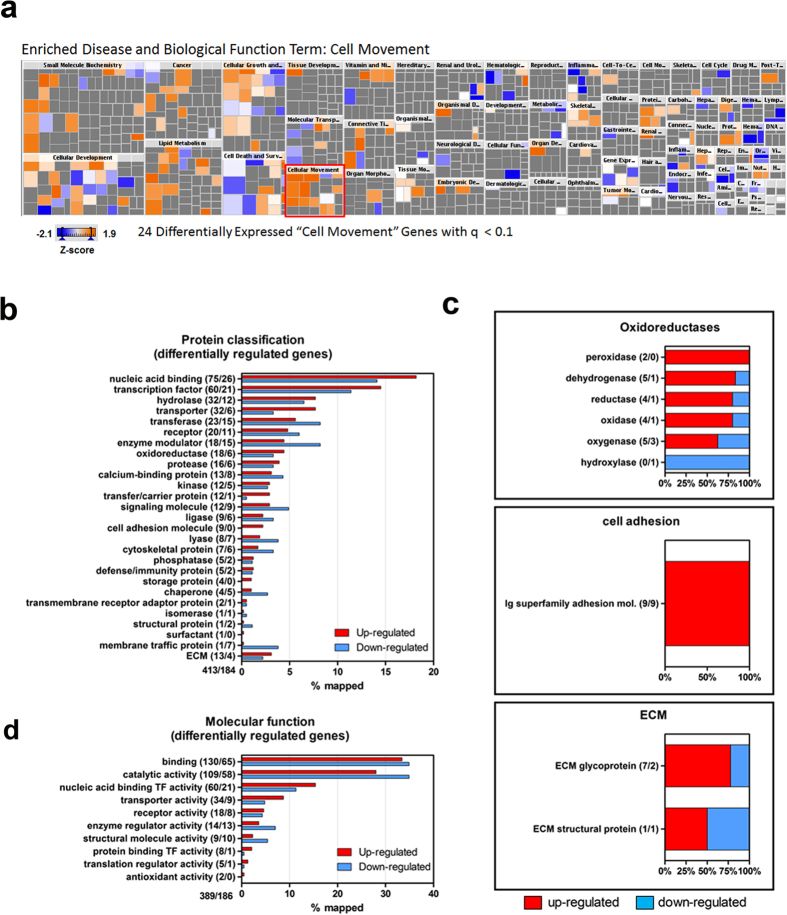
Enriched disease and biological functions and classification of differentially regulated transcripts after H_2_O_2_ treatment. (**a**) Downstream Effects Analysis in the Ingenuity’s Pathway Analysis was used to visualize, via color-coded heatmaps, putative biological and disease trends in H_2_O_2_-treated zebrafish larvae. Within the cell movement category (boxed in red) are 24 differentially expressed genes (q < 0.1, false discovery rate). The color intensity of the squares in the heatmaps reflects the strength of the absolute z-score for predictions (orange = positive, blue = negative). The categories are assembled with the most significant p-values displayed on the left of the heatmap. The size of the squares reflects the z-score values. (**b**) Protein classification of transcripts affected by H_2_O_2_ treatment of larval zebrafish. Bars represent the % of mapped transcripts to appropriate annotations. Absolute gene numbers are shown in parentheses. (**c**) Shown is the % of mapped transcripts up (red) or down (blue) regulated for three different categories: oxidoreductase, cell adhesion and ECM. (**d**) Classification of the molecular functions of affected transcripts after H_2_O_2_ treatment. Graphs represent the % of mapped transcripts to appropriate annotations. Analyses were performed using PANTHER^63^.

Knowledge-base curation of biological and molecular information facilitates the meaningful interpretation and hypothesis testing of genome-wide transcriptomic data. We applied QIAGEN’s Ingenuity^®^ Pathway Analysis (IPA) software to appraise the H_2_O_2_-regulated differentially expressed genes (Worksheet 2). Based on these analyses, we were able to generate a map of enriched biological functions after low-level H_2_O_2_ treatment. Collectively, the change in H_2_O_2_-mediated transcripts suggests an overall positive effect on cell survival, viability, growth, lipid biosynthesis and motility (Table S2). For example, IPA predicted an increase in cell survival functions after H_2_O_2_ treatment by way of induction of early pro-survival *foxo1, cxcr4, xiap* and *rictor* transcripts (Worksheet 2). Xiap is a member of the inhibitor of apoptosis family of proteins, while rictor is a subunit of mTORC2, which regulates cell growth and survival in response to growth factor and hormonal signals^60^. In addition, by using the Downstream Effects Analysis feature of the Ingenuity^®^ Knowledge Base we uncovered biological and disease trends for H_2_O_2_-treated zebrafish larvae (Fig. 3a). Using this method, a color-coded heatmap depicting z-score predictions for enriched disease and biological function terms such as “cell movement” with p-values (i.e. categories with most significant p-values are left to right of the heatmap) were generated. Importantly, a significant proportion of the “cell movement” heatmap squares contacted a positive z-score. Within the H_2_O_2_ signaling-related “cell movement” heatmap were 24 transcripts that include *mmp9, hbegf* and *adam8* (Worksheet 2). For example, Hbegf-Egfr signaling is well known to promote epithelial cell migration during development^61^. The down regulated differentially regulated transcripts after H_2_O_2_ treatment (Table S3) pointed to a decrease in cellular stress (e.g. ER stress) and cell-to-cell signaling possibly due to disruption of intercellular junctions to promote movement mediated by *cyr61* and *mylk* (Worksheet 2). Cyr61 is a secreted extracellular matrix (ECM)-associated signaling protein of the CCN family associated with improved epithelial repair^62^ and myofibroblast function in granulation tissue of wounds^3^.

### Protein and molecular function classification based on RNA-seq

We were also interested in various protein classifications specifically associated with the statistically significant H_2_O_2_-mediated differentially regulated genes. We applied the PANTHER (Protein Analysis Through Evolutionary Relationships) classification system^63^ which takes into consideration protein families, molecular functions, biological processes and pathways to facilitate the high-throughput RNA-seq data (Fig. 3b,c). The gene ontology (GO) terms used in the general protein classification are depicted in Fig. 3b and corresponding Worksheet 3. The major classifications include nucleic acid binding proteins/transcription factor elements, hydrolases, oxidoreductases, transferases, and calcium binding proteins, as examples. There were more downregulated transcripts known to code transferases, enzyme modulators, chaperones and cytoskeletal proteins. Next, these broad protein classifications were further subcategorized for more detailed evaluation (Fig. 3c). PANTHER analysis categorized 24 mapped transcripts involved in regulation of oxidoreductases in response to oxidative stress (Worksheet 4). These could reflect changes in expression value of several ROS-scavenging enzymes following H_2_O_2_ treatment. The putative increase in peroxidases is not surprising, in that for many of these enzymes the optimal substrate is hydrogen peroxide, yet it might also provide added host defense against pathogens as suggested in Table S2. Nine transcripts were mapped to the Ig superfamily of cell adhesion molecules and all found to be elevated, suggesting the potential importance of specific adhesion molecules in H_2_O_2_-mediated wound repair and migration. The ECM classification was subcategorized into ECM glycoproteins and structural proteins. Col5a3b, an alpha chain for one of the low abundant fibrillary structural collagens, was upregulated after H_2_O_2_ treatment. Itgb4, a transmembrane glycoprotein receptor that mediates cell-matrix and cell-cell adhesion and transduced signals that regulate gene expression and cell growth, was specifically increased by H_2_O_2_ treatment. Likewise, these specific protein classifications were represented functionally as depicted in Fig. 3d and Worksheet 3.

### Network of upstream regulators of H_2_O_2_ signaling in zebrafish larvae

**Figure 4:**
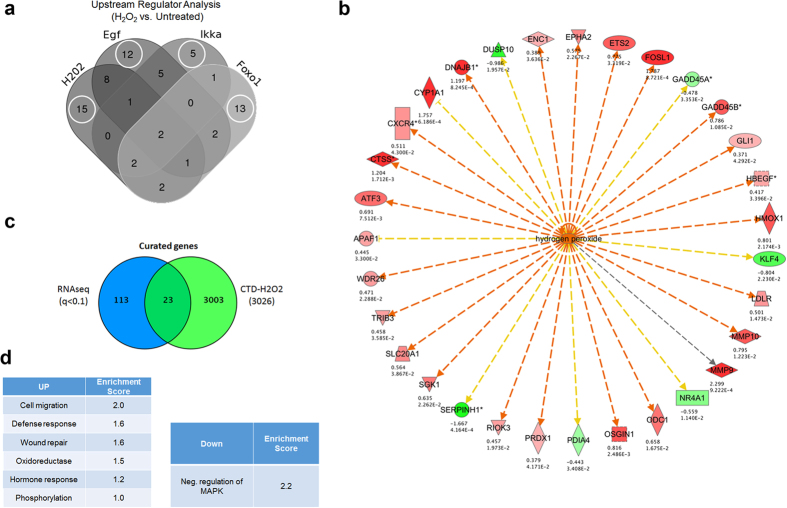
Upstream pathway analysis of larval zebrafish treated with H_2_O_2_. (**a**) Causal upstream networks determined using Ingenuity’s Upstream Regulatory Analysis after H_2_O_2_ treatment based on the literature compiled in the Ingenuity^®^ Knowledge Base. Fisher’s exact test p-values were calculated to assess the significance of enrichment of the RNA-seq data for the genes downstream of an upstream regulator (Worksheet 4). (**b**) Most significant downstream genes within the H_2_O_2_ upstream network in zebrafish samples (p < 0.05, n = 3 biological replicates). Top numerical value for each transcript represents log_2_(fold change) (H_2_O_2_ vs. untreated), while the lower value represents the p-value. Shades of red indicate the degree of upregulation, while shades of green represent the degree of downregulation. The edges connecting the nodes are colored orange when leading to activation of the downstream node, and yellow if the findings underlying the relationship are inconsistent with the state of the downstream node. Pointed arrowheads indicate that the downstream node is expected to be activated, while blunt arrowheads indicate that the downstream node is expected to be inhibited. (**c**) Overlap among the differentially expressed genes in H_2_O_2_-treated zebrafish and curated chemical-gene interactions for H_2_O_2_ derived from the Comparative Toxicogenomics Database (http://ctdbase.org). (**d**) Functional annotation analysis using DAVID^121^ of the H_2_O_2_-downstream genes. Enrichment scores ≥1 are considered significant.

H_2_O_2_ is a pleiotropic molecule capable of regulating biological systems directly or indirectly though effects on ECM components, second messengers regulating kinase-driven pathways and/or oxidation of transcription factors^14^. Given this, we attempted to elucidate the interconnecting upstream signaling and metabolic networks of H_2_O_2_ using Ingenuity’s Upstream Regulator Analysis (URA) tool^64^ (q < 0.1; Fig. 4a). This approach builds gene networks from combined gene regulatory and protein-protein mechanistic interactions. Several canonical networks (with > 1 input gene) such as H_2_O_2_, Foxo1, Egf, and Ikkα that fell within the 5^th^ percentile were assigned using URA (Worksheet 4). In brief, this analysis revealed certain effector transcripts that were common to all upstream regulators, such as *mmp9* and *hmox1*, while other transcripts were unique to a particular upstream regulator (Fig. 4a**, encircled**). For example, particular to the activation of the EGF pathway, *elf3* (Ets domain transcription factor 3) was found to be enhanced by H_2_O_2_. Elf3 is a conserved epithelial-specific transcriptional activator that is known to transactivate collagenases as well as repress pro-differentiation KRT4 (keratin 4) promoter activity^65^. The major genetic component of the overlapping networks was *hmox1* (heme oxygenase 1) (Fig. 4a). Hmox1 catalyzes the degradation of heme and displays anti-oxidative properties, and often induced in the presence of ROS intermediates^66^ to promote cell migration and proliferation in certain epithelial cell types^67^, including keratinocytes^36^. Furthermore, keratinocytes react to increased oxidative stress by induction of cytoprotective genes^68^, whereby upregulation of *hmox1* in both zebrafish and human epidermal skin cells may be a strategy for increased survival. A number of novel differentially regulated genes that were not within the overlapping H_2_O_2_-foxo1-egf-ikkα mechanistic network were identified (e.g. *ldlr, ets2, enc1, prdx1, cish, apaf1, acsl4, nes, xiap*), and may also play a role in during H_2_O_2_-induced cell migration. Through comparison of various *in vitro*-based screening methods^69^,^70^, it is still unclear if these genes are involved in injury-induced migration of keratinocytes in whole organisms as suggested by our results.

H_2_O_2_ as a major upstream regulator in larval zebrafish was investigated in more detail. H_2_O_2_ was predicted to be an upstream regulator of 31 differentially regulated genes (*UP*: *fosl1a, hmox1, mmp10, mmp9, dnajb1, epha2, odc1, osgin1, rsl1d1, atf3, ctss, cyp1a1, ets2, gadd45b, gli1, hbegf, ldlr, prdx1, riok3, sgk1, slc20a1, trib3, wdr26, apaf1, enc1; DOWN: klf4, serpinh1, dusp10, gadd45a, nr4a1, pdia4*) (Fig. 4b and Worksheet 4). These genes are predicted to belong to a H_2_O_2_-regulated pre-defined Ingenuity^®^ mechanistic network, which includes AP1, CREB1, ERK, FOS, HTT, JUN, JUND, NF-κB (complex), PPARA, PPARG, SP1, STAT3, TNF, TP53, and USF2. To further validate the data, we also assessed chemical-gene interactions for H_2_O_2_ using the Comparative Toxicogenomics Database (CTD; http://ctdbase.org). The CTD contains 3,026 curated genes for having known associations with H_2_O_2_. Of these genes, 23 (q < 0.1; Fig. 4c and Worksheet 4) were differentially regulated in H_2_O_2_-treated zebrafish larvae based on our sequencing data. Interestingly, only 4 genes overlapped with the IPA data suggesting a greater enrichment (*atf3, epha2, hmox1, odc1*). We validated the differential expression of some of these genes using quantitative PCR (Fig. 1e and Table S7). Interestingly, atf3 is an oxidative stress responsive transcription factor known to be upregulated in migrating keratinocytes after wounding^71^. Atf3 also plays a protective role in renal ischemia-reperfusion injury, and the protective mechanism may involve suppression of p53 and induction of p21 to regulate proliferation^72^. Moreover, one down regulated gene which belonged to the H_2_O_2_ network was *gadd45a*, a DNA repair enzyme that maintains genomic integrity and participates in the suppression of cancer malignancy by acting as a downstream p53 gene^73^. This indicates that H_2_O_2_ may also promote signals that counteract specific cell cycle stimuli within complex tissues and cell populations. Recently, it was shown that GADD45A blocks cell migration and invasion through altering expression of genes involved in focal adhesion, cell communication and ECM-receptor interactions^74^.

Genes that were implicated within the H_2_O_2_ upstream oxidative stress network functionally clustered into several biological categories such as cell migration, defense response and wound repair in ranking order (Fig. 4d). Genes within the cell migration cluster (*hmox1, hbegf, mmp9*/*10*) have associations with immunomodulation, energy metabolism, detoxification/cytoprotection and maintenance of the ECM in the context of injury^75^,^76^,^77^,^78^,^79^. Downstream wound repair genes include *cxcr4*, a chemokine receptor endowed with potent chemotactic activity for a number of cell types during development^80^,^81^ and under oxidative conditions such as injury/inflammation^82^,^83^,^84^ and cancer^85^. Cxcr4 is also expressed in basal keratinocytes, where it plays a role in inhibiting proliferation in the context of IL-23-mediate psoriasiform dermatitis^86^. CXCR4 and its ligand, CXCL12, were recently found to be critical downstream regulators of Ikkα-dependent non-canonical NF-κB signaling in response to a tissue-injury extracellular factor HMGB1 for mouse embryonic fibroblasts and macrophage migration^87^,^88^. Our RNA-seq data are consistent with *cxcr4* as a conserved effector of H_2_O_2_ signaling to promote wound healing. Many of these genes identified in the RNA-seq analysis were validated using conventional qPCR (Table S7).

### Low H_2_O_2_ concentrations do not alter ARE/EpRE-regulated gene expression

Oxidative stress through ROS^89^ or toxins such as mercury^90^ has been shown to stimulate binding of NFE2L1/2 (nuclear factor-erythroid 2 p45 subunit-related factor 1/2) to ARE/EpREs (antioxidant/electrophile response elements), which are found in the promoter regions of phase II detoxification enzymes and antioxidant proteins. Under homeostatic conditions, NFE2 (Nrf2) is retained in the cytoplasm by Keap1 (Kelch ECH associating protein 1) and targeted for degradation, whereas oxidative stress leads to proteasomal degradation of Keap1 and release of Nrf2 into the nucleus^89^,^91^. In contrast to Nrf2 dependency on Keap1, NFE2L1 (Nrf1) utilizes a Keap1-independent mechanism for ARE/EpRE binding^92^. Because of the important functions of Nrf1/2 in the activation of detoxifying enzymes we wanted to test whether this system is also activated in the presence of low H_2_O_2_ concentrations. Using Ingenuity’s mapping of genes to enriched canonical pathways, we first determined whether some of our identified differentially expressed transcripts have been previously associated with Nrf2/Keap1signaling (Worksheet 5). Indeed we found 9 genes that showed previous associations with this signaling pathway (*UP*: *dnajb1, fosl1a, bach1, hmox1, junb, keap1a, keap1b, sqstm1*; *DOWN*: *dnaja1, dnajc3, jund*). Only three of these genes have been also associated with oxidative stress regulation, *hmox1, jun* complex, and *sqstm1. Hmox1* activation by Nrf2 plays an important role in the arsenite-mediated oxidative stress response and jun regulation^93^. The other relationships remain to be determined.

Other factors associated with Nrf2, such as *keap1, fosl1* (an inhibitor of Nrf2), and *bach1*, which antagonizes Nrf2 binding to ARE enhancer elements under homeostatic conditions, suggest that Nrf2 signaling may be actively suppressed. To investigate this possibility, we first performed a search in our RNA-seq data for Nrf1/Nrf2 and known regulated genes^92^.To identify the correct homologs of the human *NFE2*-related genes, we first compared the human peptide sequences against the zebrafish genome using the NCBI BLAST tool. We further aligned the identified corresponding zebrafish-specific transcript sequences against the ENSEMBL zebrafish reference genome, which identified the correct ENSEMBL Gene ID as determined by the Cufflinks analysis. We found six *nfe2* genes present in the zebrafish genome, including two *Nrf1*-related genes (*nfe2l1a* and *nfe2l1b*), three *Nrf2*-related genes (*nfe2, nfe2l2a* and *nfe2l2b*), and one *Nrf3*-related gene (*nfe2l3*). Although all of these genes were expressed under homeostatic conditions, none of the *nfe2* genes were differentially regulated at a statistically significant level in the presence of H_2_O_2_ (Worksheet 1).

**Figure 5:**
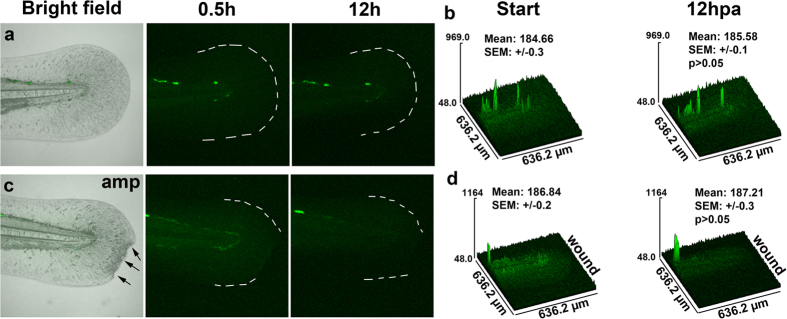
Comparison of ARE/EPRE:GFP activation in zebrafish. (**a**) The caudal fin of an uninjured EPRE:GFP larval zebrafish was imaged over the course of 12 hours. First and last images of the time-lapse sequence are shown. (**b**) Matching surface plots and quantification, comparing the fluorescence means of 4 individual fish (n = 4). Statistical significance was tested between first and last time points, showing lack of EPRE:GFP activation by 12 hours. (**c**) First and last image of a time-lapse sequence showing the amputated caudal fin (arrows) of an EPRE:GFP larval zebrafish. (**d**) Matching surface plots and quantification, comparing the fluorescence means of 6 individual fish, show that injury fails to activate EPRE:GFP.

Finally, we asked whether Nrf2 is activated upon injury in transgenic zebrafish larvae harboring an EpRE:GFP transgene^90^ using time-lapse imaging to assess changes in fluorescence after injury, which stimulates H_2_O_2_ production^21^. In support of our *in silico* predictions, we did not observe any differences in fluorescence when comparing control and injured tail fins (Fig. 5a–d). These findings suggest that ARE/EpRE-regulated genes are not significantly activated in the presence of low, wound-specific H_2_O_2_ concentrations. These results support a model in which wound-derived H_2_O_2_ stimulates the expression of genes that do not depend on Nrf2/Keap1 regulation and may only partially overlap with an oxidative stress response. We further assessed the expression of two other oxidative stress-response factors, catalase and glutathione peroxidase. The zebrafish genome harbors one *catalase* gene and eight members of the glutathione peroxidase family *(gpx1a, gpx1b, gpx2, gpx3, gpx4a, gpx4b, gpx7 and gpx8)*, none of which were significantly and differentially regulated at the mRNA level in the presence of H_2_O_2_ (Worksheet 1).

### Inference of cutaneous effects: Cross comparison of zebrafish RNA-seq and human epidermal keratinocyte microarray data upon H_2_O_2_ treatment

**Figure 6:**
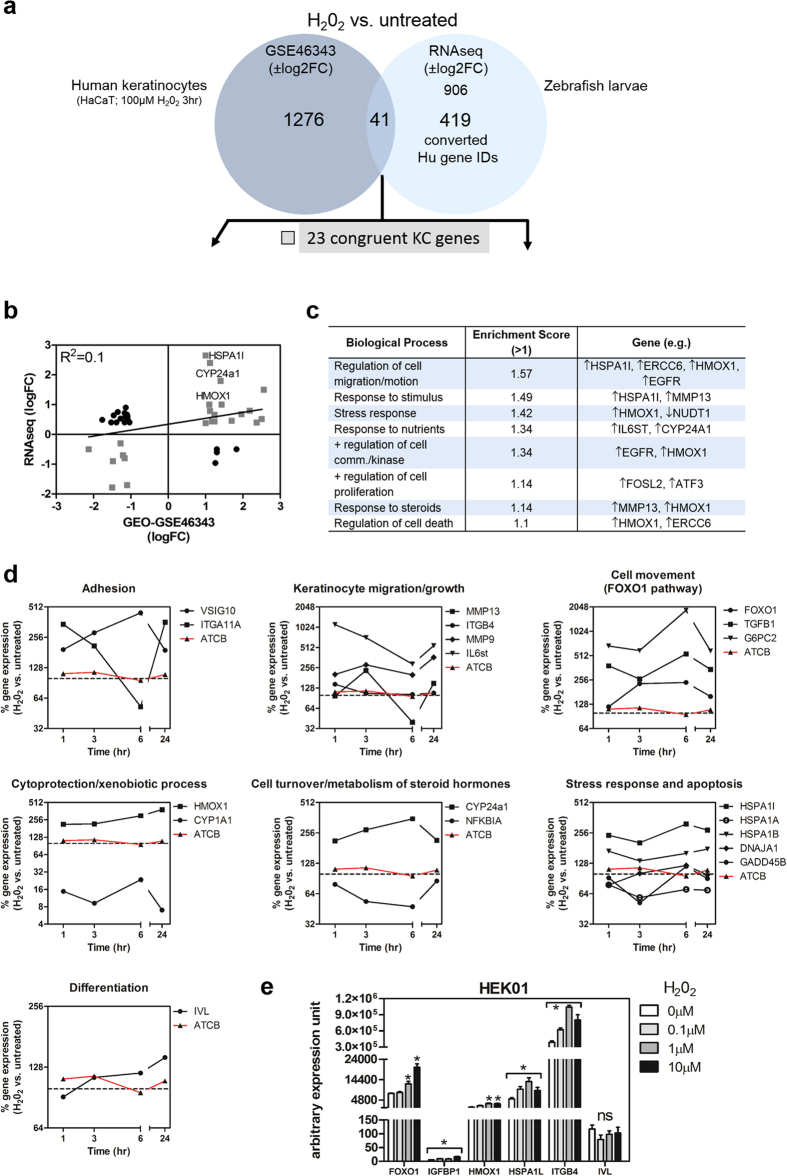
Comparative whole transcriptomic analysis between human epidermal keratinocytes and zebrafish larvae treated with H_2_O_2_. (**a**) Comparison of H_2_O_2_-mediated gene expression between HaCaT cells^36^ and zebrafish larvae reveals 23 congruent and 18 non-congruent overlapping genes. (**b**) Distribution of the overlapping genes based on logFC values. Congruent transcripts are boxed in gray. (**c**) Functional annotation clustering of the congruent genes using DAVID reveals biological processes, which are conserved between human epidermal cells and zebrafish treated with H_2_O_2_. (**d**) Detailed analysis of the HaCaT response to H_2_O_2_ [100 μM] over the entire time course within the GSE46343 study^36^. Data represented as the % gene expression of treated vs. untreated samples. (**e**) qPCR analyses of H_2_O_2_-treated HEK01 (6hr) consistent with activation of epithelial cell migration and adhesion. One-way ANOVA at an alpha = 0.05 (95% confidence interval) and Tukey’s multiple comparison post-tests were utilized. Significance is denoted with asterisks: *p < 0.05, n = 3–5 experiments. *Abbreviations: Fold change (FC), beta actin (ATCB), involucrin (IVL), integrin beta 4 (ITGB4), insulin-like growth factor-binding protein 1 (IGFBP1), heat shock 70 kDa protein 1 L (HSPA1L), not significant (ns).*

Although it is difficult to discriminate the cutaneous vs. extra-cutaneous effects of H_2_O_2_ depicted in the RNA-seq data, these studies demonstrate that H_2_O_2_ potentially activates numerous conserved gene pathways that are functionally enriched for epidermal cell migration, for example. To specifically define H_2_O_2_-induced signaling in keratinocytes, we performed a comparative analysis of our RNA-seq data with publically-available whole transcriptome microarray data using the human epithelial HaCaT cell line treated with H_2_O_2_ (GEO accession: GSE46343)^36^. This H_2_O_2_ data set was part of a larger study that was archived but has not been fully evaluated. First, we performed a comprehensive R- and Bioconductor-based normalization of the microarray data using CARMAweb^94^ (Fig. S1). In the GSE46343 study, the investigators included two untreated HaCaT samples and one H_2_O_2_-treated sample. We identified differentially regulated genes by fold-induction differences using the normalized expression values (Fig. S1c). Plots were generated to show the distribution of the Cy5 (R) against Cy3 (G) intensity ratio (M, log ratio) plotted by the average (A) intensity for each individual transcript. The red colored transcripts represent log2 ± fold change (FC) greater than twice as large or small in value compared to untreated samples. These differentially regulated genes were used for downstream applications. There were 1276 differentially regulated genes in HaCaT cells treated with H_2_O_2_ [100 μM] for 3hr compared to untreated cells (Fig. 6a and Worksheet 6). By comparing our zebrafish RNA-seq transcript set that was mapped and converted to proper human gene IDs (Worksheet 7), we identified 41 overlapping differentially regulated genes (Table S8). Among these genes, 23 were congruent by way of their directionality of gene expression (Fig. 6b). These included *egfr, hspa1l, mmp13* and *hmox1*, all implicated in cell migration and biological stress responses (Fig. 6c; Table S8 and Worksheet 8).

To further characterize H_2_O_2_ transcriptional responses in human epidermal cells, detailed gene expression analyses of human HaCaT responses to H_2_O_2_ over the entire time course in the GSE46343 study were performed (Fig. 6d). For this section, we assessed genes that characterized a specific biological or molecular process over the course of 24hr. As a control, beta actin (ATCB) levels were observed to be consistent throughout the time course after H_2_O_2_ treatment. Certain cell adhesion genes such as *Vsig10* remained elevated after H_2_O_2_ treatment. In contrast, *Itga11a* mRNA levels decreased by 6hr, and then peaked after 24hr. Genes involved in cytoprotective/xenobiotic processes such as *Hmox1* was consistently elevated, however *Cyp1a1* was suppressed throughout the time course. Based on our zebrafish RNA-seq findings, this result points to an extra-epidermal *Cyp1a1* H_2_O_2_-dependent response as it is known to be specifically enriched in intestinal tissue^95^ (Fig. 1b). For genes implicated in keratinocyte migration and growth, there was for the most part an elevated bi-phasic expression pattern over the time course. Interestingly, genes involved in the Foxo1 signaling pathway implicated as positive regulators of cell movement were all elevated after H_2_O_2_ treatment. Stress response and apoptosis factors were elevated, suppressed or exhibited no change after H_2_O_2_ treatment, suggesting a “balancing effect” to modulate stress or death responses. Importantly, we observed similar responses of both *Cyp24a1* and *Nfkbia* in HaCaT cells compared to zebrafish after H_2_O_2_ treatment (compare Figs 1f and 6d), suggesting a conserved role for H_2_O_2_ on members that regulate cell turnover and metabolism of steroid hormones. In addition, we only observed minimal effects on crucial indicators of keratinocyte differentiation (e.g. involucrin) over the time course (Fig. 6d). In order to further corroborate the H_2_O_2_-mediated gene expression profile not only observed in HaCaT cells but in our zebrafish RNA-seq data as well, we utilized another human epidermal keratinocyte line (HEK01) which retains normal epidermotropic responses^96^ (Fig. S2) in RT-qPCR studies. We show statistically significant transcriptional increases in cell migration and growth factor binding proteins such as *Itgb4* (integrin beta 4) and *Igfbp1* (insulin growth factor binding protein 1) in HEK01 cells treated with H_2_O_2_ respectively (Fig. 6e). These transcripts were also elevated in our zebrafish RNA-seq data (Worksheet 1). Furthermore, we observed increases in *Foxo1, Hmox1*, and *Hspa1l* (heat shock 70 kDa protein 1 L), but not in *Ivl* (involucrin) message levels after H_2_O_2_ treatment, suggesting a cytoprotective and pro-migratory cellular phenotype. Overall, many of the genes (e.g. *hmox1, cyp24a1, mmp13, il6st, hspa1l*) from the zebrafish RNA-seq studies followed similar expression patterns when compared to human epidermal cells treated with H_2_O_2._

**Figure 7:**
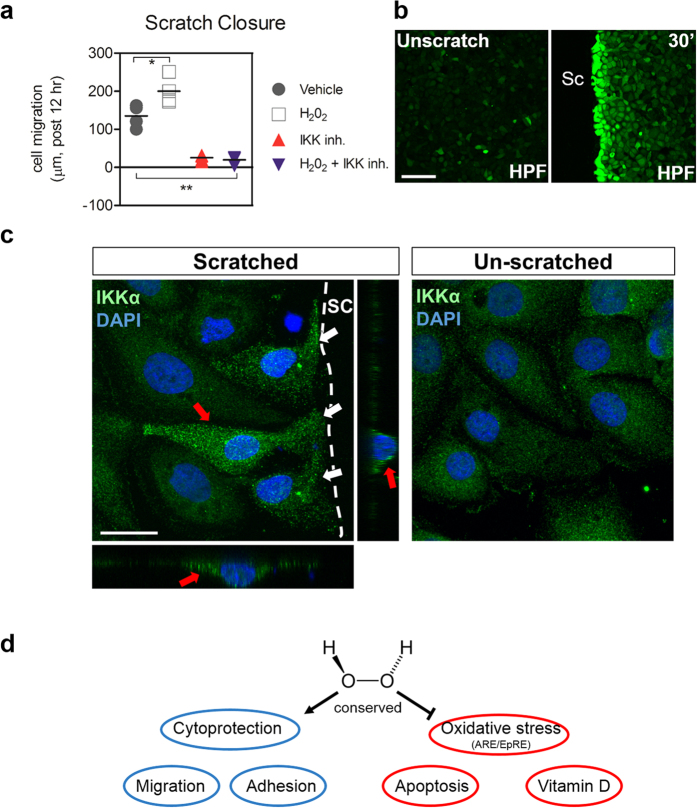
Inhibition of IKK/Ikk delays scratch closure and intracellularly accumulates within injured epidermal keratinocytes. (**a**) IKK is necessary for H_2_O_2_-induced HEK01 keratinocyte migration after wounding. Scratch assays were performed with H_2_O_2_ (0.1 μM) and Wedelolactone (50 μM) using HEK01 cells. Two-way ANOVA at an alpha = 0.05 (95% confidence interval) and Bonferroni’s multiple comparison post-tests were utilized. Significance is denoted with asterisks: *p < 0.05, **p < 0.01 (n ≥ 3–5 cell culture experiments). (**b**) Rapid H_2_O_2_ production using 1 μM HPF (hydrogen peroxide fluorogenic probe) at the scratch (sc) margin of HEK01 keratinocytes within 30 minutes. Bar = 100 μm (**c**) Rapid subcellular accumulation of IKKα within injured HEK01 cells at the scratch (sc) margin (white arrows) after 30 minutes compared to unscratched cells. Orthogonal views (red arrows) of an injured cell show peri-nuclear and cytoplasmic distribution and accumulation of IKKα. Bar = 20 μm. (**d**) Schema of overall findings.

Lastly, in order to provide evidence of cell-autonomous signaling conservation, we utilized HEK01 keratinocytes to pharmacologically inhibit IKK, one of the major upstream pathways predicted to be activated by H_2_O_2_ in zebrafish. While we observed accelerated gap closure when wildtype HEK01 cells were treated with low (0.1%) H_2_O_2_ (Fig. 7a), scratch-induced migration was blocked when cells were treated with the IKK kinase inhibitor Wedelolactone, even in the presence of H_2_O_2_. Thus IKK signaling appears to be downstream and dependent on H_2_O_2_. To corroborate these findings, we monitored H_2_O_2_ and IKKα localization after scratch injury. H_2_O_2_ was specifically and rapidly detected via a chemical H_2_O_2_ sensor in injured keratinocytes at the scratch margin (Fig. 7b). Similarly, anti-IKKα immunofluorescence staining revealed increased accumulation of IKKα in picnotic peri-nuclear and cytoplasmic subcellular domains specifically within keratinocytes at the scratch wound margin (Fig. 7c). These findings indicate that IKK may play an important functional role in response to injury, which corroborates our inhibitor results. Collectively, our findings provide support of the conserved wound repair-promoting functions of H_2_O_2_ in the epidermal cells (Fig. 7d).

## Discussion

A plethora of recent scientific findings strengthens the role of H_2_O_2_ in signal transduction within human and murine systems^97^. Within both a mouse wound healing model^24^ and human HaCaT keratinocyte culture system^26^, H_2_O_2_ has been shown to increase keratinocyte viability and migration after injury. Likewise within non-mammalian vertebrate systems, such as zebrafish, findings suggest that H_2_O_2_ is a crucial second messenger for growth factors and cytokines in the regeneration of axons and the recruitment of leukocytes to the wound during repair^20^,^21^,^23^. Our RNA-seq data of zebrafish larvae treated with low concentrations of H_2_O_2_ suggests that the activation of conserved pro-survival and migratory pathways are in agreement with findings from higher organisms. Interpretation of the RNA-seq data is difficult to assess without tissue- and cell-type specific filtering strategies, although much of the responses appear to be epithelial in nature (Fig. 1b). For example, in the context of tail fin amputation, sustained ROS signals can activate both apoptotic and proliferative pathways necessary for blastema formation and tissue regeneration when appraised collectively^98^. In addition, certain cutaneous cell types such as damaged sensory nerves, which exhibit their own unique transcript profile, help activate keratinocyte migration by the release of specialized trophic factors^99^. For this reason, we attempted to stratify keratinocyte and extra-epidermal effects by comparing our H_2_O_2_-treated zebrafish findings with a human HaCaT microarray study. We are also aware that H_2_O_2_ signal transduction is largely mediated through post-translational modifications (PTMs) of target proteins such as kinases that can regulate downstream transcription factors^15^. These H_2_O_2_-related PTMs are capable of oxidizing, unfolding and inactivating or activating certain types of proteins like kinases and tyrosine phosphatases within catalytic domains to regulate downstream cascades^100^. Yet the goal of this study was not to study these post-translational events *per se*, but rather the relevance of those downstream transcriptional targets which may be modulated by putative post-translational influences, such as upstream activity of IKKα, as identified in our study (Fig. 5a).

In this study we provide a comprehensive overview of the genome-wide effects of H_2_O_2_ treatment of larval zebrafish using next generation sequencing. Given the known positive effects of low H_2_O_2_ levels on injury-induced cell migration, we sought to identify novel sets of genes associated with H_2_O_2_ treatment alone. In an unbiased manner through functional annotation and upstream regulator analyses, we observed cell migration and survival pathways to be highly enriched in our zebrafish RNA-seq data. For example, we identified novel ECM regulatory factors that may play a role in H_2_O_2_-induced cell migration (Fig. 3c and Worksheet 3). It is known that interstitial reserves of collagenases, proteinases and plasminogen activator within epidermal cells is necessary to degrade the ECM for active migration during repair, and we provide evidence of other factors which may regulate this specific process. We also identified sets of adhesion factors regulated by H_2_O_2_, which may feed into known keratinocyte integrin receptors commonly overexpressed after injury. These receptors function to ligate with newly synthesized and processed basement membrane proteins adjacent to the wound margin for proper anchorage of cells^101^,^102^. Further studies are required to characterize our identified factors as they relate to tissue injury.

From the analysis of our zebrafish RNA-seq data it became obvious that low levels of H_2_O_2_ promote an overall beneficial outcome. The Foxo1 downstream genes involved in lipid metabolism that were up regulated after H_2_O_2_ treatment include *g6pc, gpam,* and *pck1*, indicative of activation of a metabolic process to support energy-demanding activities such as cell migration (Worksheet 4). Importantly, the effects of H_2_O_2_ on the Foxo1 and TGFβ signaling pathways to promote cell migration appear to be conserved between zebrafish and human epidermal cells (Figs 4a and 6d,e). Upregulated genes involved in cell communication, i.e. signaling or attachment between another cell and ECM, include *rictor, traf6* and *hmox1*. Rictor is part of the mTOR complex, and controls cell growth and survival via the actin cytoskeleton^103^. Importantly, activation of the mTOR signaling pathway plays a positive role during wound repair, as recent studies have shown that epithelial-specific ablation of *Pten* and *Tsc1* (inhibitors of mTOR) can further increase epithelial cell migration and wound healing^104^.

We identified a set of enriched vitamin D signaling pathway genes (*cyp24a1, cebpb, igfbp1, klf4*) that were influenced by H_2_O_2_ treatment (Worksheet 5). As mentioned previously, upregulation of *cyp24a1*, which is responsible for 1,25D_3_ VDR ligand decomposition, suggests suppression of the vitamin D system by H_2_O_2_ in both zebrafish and human skin cells (Fig. 6). Interestingly, *klf4* (kruppel-like factor 4) message was significantly downregulated after H_2_O_2_ treatment (Fig. 4b and Worksheet 5). KLF4 is a putative tumor suppressor and known epidermis-enriched transcription factor that facilitates the differentiation of epidermal layers^105^. Importantly, it was previously shown that 1,25D_3_-mediated induction of KLF4 within human epidermal keratinocytes supports the differentiation and barrier functions of the skin^106^. Therefore based on previous and our current findings, it is likely that H_2_O_2_ converges on the vitamin D genomic network in epidermal keratinocytes through *KLF4* during H_2_O_2_-induced cell migration. It is currently unclear whether *KLF4* is a direct transcriptional target of the VDR in keratinocytes.

With regard to cell proliferation in the context of tissue injury, the role of H_2_O_2_ is unclear. In one study, *in vitro* scratch assays using injured keratinocytes showed limited proliferative effects based on genome-wide microarray analysis^69^. H_2_O_2_ has been detected in mouse wound sites^24^,^107^ and edges^25^ and its elimination by catalase over-expression in mice delays wound closure^25^. Migrating keratinocytes of acute wounds track behind proliferative keratinocytes *in vivo* and revert back to their original differentiated phenotype toward proper wound closure^6^. H_2_O_2_ treatment is known to result in EGFR phosphorylation, as well as phosphorylation of the ERK1/2 cell stress transducer which is a mitogen-activated protein kinase (MAPK) member^26^, but the *in vivo* context is unknown. Within the Egf upstream network, the downstream upregulated cell migration-related genes (*cxcr4, hmox1, mmp9*) overlapped with many of the H_2_O_2_-related genes, yet the underlying cross-talk relationships remain unknown. For example, within the anti-apoptosis gene cluster, the STAT inhibitor, *socs3*, was upregulated after H_2_O_2_ treatment, which is consistent with increased cxcr4 to potentially block JAK/STAT3-mediated cell proliferation and growth involved in a negative feedback loop^86^. Furthermore, EGF was shown to promote tyrosine phosphorylation of SOCS3 which inhibits JAK/STAT signaling^108^ to potentially ensure keratinocyte migration through the interactions with the Ras pathway^109^,^110^. How our newly identified H_2_O_2_-mediated anti-apoptotic factors are involved in the tissue repair process will require further studies as well.

In this study, we showed that H_2_O_2_-mediated activation of NF-κB was conserved in zebrafish larvae as in other mammalian model systems, yet how H_2_O_2_ activates NF-κB is not fully understood^111^. As previously mentioned, NF-κB activation by H_2_O_2_ can occur through multiple mechanisms and cell types^55^,^56^,^57^. This would include, based on our results, the downregulation of *nfkbiaa* (IκBα homologue) transcript. NF-κB resting activity is generally maintained through its cytoplasmic associations and sequestration with inhibitor proteins such as IκBα^112^. Classically, in order for NF-κB to become activated, IκBα is phosphorylated and ubiquitinated so that the nuclear localization signals are exposed for NF-κB’s translocation. Another caveat to the upstream regulation of NF-κB activity is that phosphorylation of IκBα is catalyzed by IκBα kinase (IKK) which comprises of IKKα, IKKβ and IKKγ^112^. It is known that H_2_O_2_ can activate IKKs in certain cell types such as murine fibroblasts^113^, the consequence of which may lead to increased phosphorylation of catalytic IKK subunits^114^. Alternatively, it is possible that H_2_O_2_ may regulate kinases upstream of IKK to modulate NF-κB activity^111^. Interestingly based on our RNA-seq data, IKKα was predicted to be a major upstream regulator of H_2_O_2_ signaling. Furthermore, we observed intracellular cytoplasmic and perinuclear accumulation of IKKα within injured human keratinocytes, which are also known to exhibit increased H_2_O_2_ levels upon wounding. This observation may suggest key IKK interactions and activities in specific intracellular domains during injury. Also, this feature is unique to IKKα’s known nuclear function during epidermal differentiation and barrier roles in the skin^115^. In the differentiated epidermis, IKKα has additional kinase- and NF-κB-independent nuclear repressor functions to maintain skin homeostasis^115^,^116^,^117^,^118^. Importantly, inhibition of functional IKK attenuated keratinocyte migration suggesting a positive role of cytoplasmic and peri-nuclear IKKα. Therefore, it is likely that H_2_O_2_ converges on combined upstream post-translational and downstream genomic NF-κB activating pathways that occurs through rapid IKKα activation and *nfkbiaa*/*IκBα* transcriptional downregulation within epidermal cells, respectively. It is our contention that low H_2_O_2_ levels favor NF-κB signaling to promote increased cellular survival, as there are few examples where NF-κB contributes to cell death^119^, supporting the overall cytoprotective theme. Furthermore, IKKα may have alternative distinct targets besides the NF-κB system upon H_2_O_2_ treatment, which will require future investigations.

## Materials and Methods

The study was carried out in accordance with the approved NIH guidelines.

### Zebrafish husbandry

Zebrafish (*nacre* strain) were bred and raised according to established protocols. All efforts were made to minimize suffering, using a 1:1000 dilution of 2-phenoxyethanol for anesthesia and 1:500 dilution of 2-phenoxyethanol for euthanasia. Zebrafish embryos and larvae were handled in strict accordance with good animal practice as approved by the appropriate committee (MDI Biological Laboratory animal core IACUC number 13–20). This study was approved by the National Human Genome Research Institute Animal Care and Use Committee, MDIBL Institutional Assurance # A-3562-01 under protocol # 14-09. Embryos were kept on a 14:10hr light/dark cycle at 28.5 °C and maintained in Ringers solution. All efforts were made to minimize suffering, and tricaine was used for euthanasia.

### H_2_O_2_ treatment, RNA isolation and preparation for RNA-sequencing

Pools of about 500 embryos for each of three biological replicates were derived from 5 independent mating pairs and raised under separate conditions to larval stages until 4 dpf. Larvae were anesthetized in Tricaine (Sigma-Aldrich, USA) and treated for 3 hours with 3 mM (0.01%) H_2_O_2_. All larvae were subsequently homogenized in Trizol (Life Technologies, USA) and total RNA was extracted using a chloroform extraction protocol and treated with DNAse. Messenger RNA (mRNA) was subsequently purified from total RNA using biotin-tagged poly-dT oligonucleotides and streptavidin-coated magnetic beads (mRNA Seq Sample Prep kit; Illumina Inc.), followed by quality control using an Agilent Technologies 2100 Bioanalyzer (values > 7 were used for sequencing). The poly(A)-tailed mRNA samples were fragmented and double-stranded cDNA generated by random priming for deep sequencing studies.

### Library preparation and sequencing for RNA-seq

To generate each bar-coded RNA-seq library, the ends of the fragmented cDNA were converted into phosphorylated blunt ends. An ‘A’ base was added to the 3′ ends and Illumina^®^-specific adaptors were ligated to the cDNA fragments. Using magnetic bead technology, the ligated fragments were size-selected and a final PCR was performed to enrich the adapter-modified cDNA fragments using primers annealing to the adaptors. In this approach, only the cDNA fragments with adaptors at both ends were amplified. Sequencing libraries were validated using an Agilent Technologies 2100 Bioanalyzer to characterize cDNA fragment sizes. The concentration of cDNA fragments with the correct adapters on both sides was then determined using a quantitative PCR strategy (KapaBiosystem, Cambridge, MA). Paired-end sequencing was performed on the Illumina^®^ HiSeq2000 using a sequencing-by-synthesis process. To minimize sequencing batch effects, all samples were bar-coded. Barcoded sequencing libraries representing all six samples were multiplexed and sequenced on a single lane of an IlluminaHiSeq2000 using manufacturer’s protocols. A total of 246,647,690 pairs of 100 bp paired-end sequences were generated with each sample having between 37.6–49.8 million pairs per library. RNA-seq data has been submitted to NCBI (GEO accession: GSE75728).

### Mapping, quantification and classification analyses of RNA-seq data

Sequence quality was assessed using Fastqc QC (v0.5; http://www.bioinformatics.babraham.ac.uk/projects/fastqc) and trimmed using Trimmomatic (v. 0.32)^120^. RSEM (v 1.2.16)^39^ was used to align paired-end sequence reads to transcript annotated by Ensembl (v. 73) from the zebrafish genome (Zv9). Transcript abundance, expressed as read counts per million (CPM), were analyzed using R/edgeR (v. 3.8.0)^40^. Pearson correlation was calculated between samples to examine within and between group variation of log_2_(CPM) values in R (v. 3.1; http://r-project.org). Down/upstream/enriched pathway and functional classification analyses were performed using a combination of programs (PANTHER v9.0^63^; DAVID^121^) with mapped genes. Data were analyzed through the use of QIAGEN’s Ingenuity^®^ Pathway Analysis (IPA^®^, QIAGEN Redwood City, www.qiagen.com/ingenuity).

### Quantitative RT-PCR

Total RNA was purified using the Qiagen RNeasy mini kit (Qiagen, Valencia, CA). RNA was reversed-transcribed using Superscript reverse transcriptase (Invitrogen) and random hexamer primers. Gene expression was normalized with actin/18sRNA mRNA and analyzed using the comparative CT Livak method (Livak, Schmittgen 2001) using Brilliant II SYBR^®^ Green qPCR Master Mix (Agilent). Primers used are listed in Table S4.

### Cross comparison of Zebrafish RNA-seq and human HaCaT (keratinocyte) genome-wide microarray data

H_2_O_2_-mediated differentially regulated genes in human skin epithelial keratinocytes (HaCaT) were determined from publicly available gene expression data^36^ (GEO accession: 46343; www.ncbi.nih.gov/geo) using Comprehensive R based Microarray Analysis (±log_2_FC)^94^. The IDs of genes which were differentially regulated in zebrafish after H_2_O_2_ treatment were converted to human gene IDs using DAVID. Not all zebrafish larval transcript names could be converted to a human orthologue.

### H_2_O_2_ treatment, imaging, and data analysis of wildtype, EPRE:GFP and Tg(NF-κB:EGFP) zebrafish

Four days post fertilization (dpf) wildtype larvae were treated with pentafluorobenzenesulfonyl-fluorescein (HPF) and 0.01% (3 mM) H_2_O_2_ or with HPF alone for 3 hours and static images were recorded using a FV1000 (Olympus) confocal microscope. Uninjured and amputated caudal fins of 3 dpf EPRE:GFP larvae were recorded in 12hr time-lapse movies (one stack every 30 min) on a FV1000 (Olympus) confocal microscope. Three dpf larvae of the Tg(NF-κB:EGFP) reporter strain^59^ either untreated or treated with 0.01% (3 mM) H_2_O_2_ were imaged using a FV1000 (Olympus) confocal microscope. Both whole larvae (upper) and tail fins were imaged separately at the beginning and end (2hr post treatment) of an experiment. Quantitative analysis using relative mean fluoresces of the z-stack projected images using ImageJ were performed from three independent experiments using Surface Plot and Measurement tools.

### Human keratinocyte (HEK01) scratch wound, inhibition and immunofluorescence assays

HPV-16 transformed human epidermal keratinocytes (HEK01; ATTC, CRL-2404) were maintained in keratinocyte-serum free (KSF) medium (Gibco-Brl 17005-042) supplemented with 5 ng/ml human recombinant EGF, low CaCl_2_ (0.06 mM) and 2 mM L-glutamine. Cells were incubated with 8% CO_2_ and 92% humidified atmosphere at 34 °C, and seeded (4 × 10^4^ cells/cm^2^) in glass bottom tissue culture plates pre-coated with type I collagen (Gibco-Brl, R-011-K). The scratch assay was used to evaluate cell migration and wound recovery (Goetsch KP, Niesler CU, 2011). Cells were grown to confluence, replaced with EGF-minus media for 12hr, refreshed with complete media and glass Pasteur pipette tips were used to make vertical scratches with maximal diameters along the surface of the vessels. Wells were immediately washed with PBS to avoid re-plating of disassociated cells. Migration of cells was documented with time-lapse imaging immediately after scratching using a motorized/heated stage, and the average change in migration distance was calculated using >16 lines (grid) spanning scratch margins distributed equally. HEK01 were pre-treated for 15 min with the IKK inhibitor at concentrations of 50 μM. IKK wedelolactone (Millipore, 401474; IC_50_ = 10 μM) inhibitor was kept as a stock solution in DMSO. For immunofluorescence staining, HEK01 were fixed in 4% PFA (15 min) and permeabilized in 0.25% triton-X (10 min) at room temperature. HEKs were blocked with 1%BSA/10% goat serum/0.1% Tween-20 in PBS (30 min) and incubated with primary antibodies (IKKα, Abcam, ab4111; 1:200–500) in blocking buffer overnight at 4 °C. Cells were washed and then labeled with AlexaFluor^®^488 anti-rabbit IgG (Molecular Probes, Invitrogen) and DAPI. Laser confocal scanning microscopy images were obtained using an inverted Olympus FV1000 confocal microscope. A series of three-dimensional “z-axis” image projections of entire cell axial depths were obtained in XYZ scan mode set to 1–3 μm/slice and a sample speed of 12.5 (μs/pixel) to obtain orthoganol views. HEK01 were pretreated for 30 min with the H_2_O_2_ sensor (HPF, Millipore, cat. nr. 386794; C_28_H_13_F_5_O_8_S or acetyl, pentafluorobenzenesulfonyl fluorescein), scratched and then detected. Briefly, a 473-nm laser beam was used to epi-illuminate a H_2_O_2_ sensor kept in DMSO (vehicle). In initial experiments, the optimal signal:noise ratio was empirically determined (1 μM).

### Statistical analyses

GraphPad Prism version 4.0 (GraphPad Software Inc., San Diego, CA, U.S.A.) was used for statistical analyses. Data were analyzed using t-test for comparisons of two or one-way ANOVA for comparisons of groups equal to or greater than three. *P* ≤ 0.05 was considered significant.

## Additional Information

**How to cite this article**: Lisse, T. S. *et al.* Comparative transcriptomic profiling of hydrogen peroxide signaling networks in zebrafish and human keratinocytes: Implications toward conservation, migration and wound healing. *Sci. Rep.* **6**, 20328; doi: 10.1038/srep20328 (2016).

## Supplementary Material

Supplementary Information

Supplementary Worksheet 1

Supplementary Worksheet 2

Supplementary Worksheet 3

Supplementary Worksheet 4

Supplementary Worksheet 5

Supplementary Worksheet 6

Supplementary Worksheet 7

Supplementary Worksheet 8
