# Supplementary material for: Comparative transcriptomic profiling of hydrogen peroxide signaling networks in zebrafish and human keratinocytes: Implications toward conservation, migration and wound healing
Source: Sci Rep. 2016 Feb 5;6:20328. doi: 10.1038/srep20328 (PMC4742856; doi:10.1038/srep20328)
Supplement: Supplementary Information [file srep20328-s1.pdf]

Table S1. Top and Bottom 10 most up- and down-regulated statistically significant transcripts (H_2O_2 vs. untreated zebrafish larvae)

Rank	Symbol	logFC	P-value	Description	GO Slim (summary)
**Up-regulated**					
1	hsp70l	2.6	0.030	heat shock cognate 70-kd protein, like	cell morphogenesis; anatomical structure development; cell cycle; ion binding; response to stress
2	mcm5	2.5	0.004	MCM5 minichromosome maintenance deficient 5	cell morphogenesis; anatomical structure development; cell cycle
3	mmp9	2.2	0.007	matrix metalloproteinase 9	peptidase activity; ion binding
4	mmp13a	1.9	0.025	matrix metalloproteinase 13a	peptidase activity; ion binding; cell motility; locomotion; anatomical structure development; embryo development
5	cyp24a1	1.8	0.038	cytochrome P450, family 24, subfamily A, polypeptide 1	oxidoreductase activity; ion binding
6	fosl1a	1.7	0.016	FOS-like antigen 1a	nucleic acid binding transcription factor activity; nucleus
7	cyp1a	1.7	0.030	cytochrome P450, family 1, subfamily A	oxidoreductase activity; ion binding
8	cry5	1.7	0.033	cryptochrome 5	lyase activity; cellular nitrogen compound metabolic process; response to stress; DNA metabolic process
9	plek2	1.5	0.007	pleckstrin 2	lipid binding; signal transduction; ion binding
10	per2	1.4	0.006	period homolog 2	signal transduction; response to stress
**Down-regulated**					
**Rank**	**Symbol**	**logFC**	**Pvalue**	**Description**	**GO Slim (summary)**
1	npas4a	-2.2	0.047	neuronal PAS domain protein 4a	DNA binding; signal transduction; organelle; nucleus; biosynthetic process; cell; intracellular; cellular nitrogen compound metabolic process
2	plekhf1	-1.8	0.044	pleckstrin homology domain containing, family F	lipid binding; ion binding
3	serpinh1b	-1.6	0.027	serpin peptidase inhibitor, clade H, member 1b	enzyme regulator activity; cytoplasm; response to stress
4	EGR4	-1.5	0.045	early growth response 4	ion binding
5	nfκbiaa	-1.4	0.042	nuclear factor of kappa light polypeptide gene enhancer in B-cells inhibitor, alpha a	DNA binding, inhibitor of NF-κB
6	lrata	-1.2	0.008	lecithin retinol acyltransferase a	NA
7	fabp11b	-1.2	0.019	fatty acid binding protein 11b	lipid binding; transport
8	GPR3	-1.1	0.007	G protein-coupled receptor 3	signal transduction
9	noxo1a	-1.1	0.017	NADPH oxidase organizer 1a	lipid binding; ion binding
10	elf3	-1.1	0.022	E74-like factor 3	nucleic acid binding transcription factor activity; nucleus

Table S2. Functional annotation (Enriched Pathways) of RNA-seq data: Up-regulated differentially regulated gene set of H_2O_2 vs. untreated larval zebrafish

**<u>Up-regulated Gene Set</u>** **IPA Category and Predicted Activation**	**p-Value**	**# Molecules**
Cellular Growth and Proliferation	1.25E-05	105
Cell Death and Survival	1.11E-05	84
Organismal Survival (↓death)	5.54E-04	76
Gene Expression	5.79E-07	75
Cellular Development (↑differentiation)	8.01E-06	66
Tissue Morphology (↑cell quantity)	5.39E-05	56
Molecular Transport	9.67E-03	46
Cell Death and Survival (↑survival)	9.31E-04	45
Cellular Function and Maintenance (↑homeostasis)	1.14E-03	43
Cell Death and Survival (↑viability)	1.11E-03	42
Small Molecule Biochemistry (lipid amount)	2.63E-05	35
Lipid Metabolism (↑synthesis)	3.28E-05	32
Organismal Development (↑size)	7.99E-03	31
Gene Expression (transactivation)	8.92E-06	30
Cellular Development (↑connective tissue diff.)	4.03E-06	28
Cellular Movement	7.72E-03	24
Immune Cell Trafficking	7.72E-03	24
Tissue Morphology (connective tissue)	2.04E-04	23
Digestive System Development and Function	2.42E-03	23
Cellular Development (prolif. immune cells)	4.38E-03	23
Molecular Transport (steroids)	5.31E-05	22
Cellular Movement (↑invasion)	1.50E-03	22
Lipid Metabolism (fatty acids)	1.57E-03	21
Embryonic Development (↑abdomen)	5.31E-03	20
Hematological System Development (↑cell movement)	4.70E-03	17
Organ Morphology (skin)	3.21E-04	14
Vitamin and Mineral Metabolism (↑terpenoid)	6.30E-04	14
Cell Signaling	4.32E-04	14
Vitamin and Mineral Metabolism (↑steroids)	7.55E-04	13
Free Radical Scavenging	1.18E-04	11
Hair and Skin Development and Function (epidermal cells)	1.27E-03	9
Connective Tissue Development and Function (fibroblasts)	1.02E-02	8
Cellular Movement (fibroblasts)	1.07E-02	8
Hepatic System Development (regeneration)	6.05E-05	7
Organismal Injury (↓glomerulonephritis)	8.18E-03	7
Hair and Skin Development (keratinocytes)	1.24E-02	7
Vitamin and Mineral Metabolism (quantity)	2.22E-03	6
Antimicrobial Response	2.88E-03	6
Immune Cell Trafficking (lymphocytes)	5.01E-03	6
Tissue Development (adhesion)	1.37E-02	5
Vitamin and Mineral Metabolism (alitretinoin)	1.09E-02	2

Note: Predicted direction (activation/deactivation) shown by arrows; Table corresponds to Worksheet 2.

Table S3. Functional annotation (Enriched Pathways) of RNA-seq data: Down-regulated differentially regulated gene set of H_2O_2 vs. untreated larval zebrafish

**Down-regulated Gene Set**<br>**IPA Category and Predicted Activation**	**p-Value**	**# Molecules**
Inflammatory Response	3.34E-02	21
Cellular Compromise (ER stress)	1.51E-06	10
Organ Morphology (abnormal morphology of gland)	3.03E-02	9
Molecular Transport	2.82E-02	7
Organ Morphology (exocrine cells)	2.82E-05	6
Organismal Injury (↑oligozoospermia)	4.63E-03	6
Cell Morphology (abnormal morphology of epithelial cells)	5.16E-03	6
DNA Replication, Recombination, and Repair	2.74E-02	6
Tissue Morphology (abnormal glandular epithelium)	1.55E-04	5
Tissue Development (organization of ECM)	7.08E-03	5
Inflammatory Response (antigen presenting cells)	2.54E-02	5
Cellular Function and Maintenance (phagocytosis)	1.28E-02	4
Cell Death and Survival (B lymphocytes)	2.19E-02	4
Protein Folding	3.11E-02	4
Small Molecule Biochemistry (cholesterol hydroxylation)	2.94E-03	2
Visual System Development and Function (abnormal morphology)	1.02E-04	3
Free Radical Scavenging (induction of ROS)	1.79E-02	2
Post-Translational Modification (refolding)	1.79E-02	2
Lipid Metabolism (catabolism of steroid)	2.42E-02	2
Cell-To-Cell Signaling (disruption of intercellular junctions)	2.42E-02	2
Free Radical Scavenging (metabolism of superoxide)	3.11E-02	2

Note: Predicted direction (activation/deactivation) shown by arrows; Table corresponds to Worksheet 2.

Table S4: Primers

Up-regulated genes			
		Zebrafish (RT-PCR)	
primer ID	primer sequence	gene name	product length
junbb_fwd	AGTCGGAGCCTCCCATTTAC	Jun B proto-oncogene	400bp
junbb_rev	AGAACAGACGCTGTGTTGGA		
xpc_ex8_fwd	ATGGAGCCGTGAAAATATGC	Xeroderma pigmentosum	396bp
xpc_ex8_rev	TAGTCTCCTCCCACCACTCG		
scd_ex6_fwd	CCACACGTTTCCCTACGACT	Stearoyl-Desaturase	397bp
scd_ex6_rev	GAGAAGAGGAAGCCGGAAAC		
cyp1a_ex2_fwd	GTCCTGGAAATCGGAAACAA	Cytochrome P450 Family 1, Subfamily A polypeptide	398bp
cyp1a_ex2_rev	AGCTTCCATCGGCTTTCATA		
sqstm1_ex3_fwd	TCACTTTAGCCCTCCTGGTC	Sequestosome 1	396bp
sqstm1_ex3_rev	CCCACATGCAGTGTCTCATC		
odc1_ex2_fwd	CGCGGTTTACCACAGCTACT	Ornithine Decarboxylase	402bp
odc1_ex3_rev	CAGGAAGGCGAAGTCAAAGT		
mmp13a_ex2_fwd	AATTACCTGACTCGACTGTATGG	Matrix Metalloproteinase 13a	404bp
mmp13a_ex3_rev	CCAGTGGCGAAGAAGATCA		
apold1_ex14_fwd	CATCATCATCTCTGCCATCG	Apolipoprotein L domain containing 1	395bp
apold1_ex15_rev	AGCCCTTCTTCACCTCCTTC		
plek2_ex9_fwd	ATCGCTCTCTGAATGGCACT	Pleckstrin 2	392bp
plek2_ex9_rev	TGCATTCAGATTAACGCACA		
fosl1a_ex1_fwd	TTTTTATTTTATTCTACCGCTGTCA	Fos-like antigen 1	390bp
fosl1a_ex1_rev	CTGTTGTTGTTGGGTTGTGC		
egfr_ex1_fwd	AACGGATTCCAGGTTTTTCC	Epidermal growth factor receptor	400bp
egfr_ex4_rev	GTCTGGGTTATCCAGGTTGG		
mknk1_ex14_fwd	GTCTGCTCCATGAGGCTTTC	MAPK-interacting serine/threonine kinase 1	398bp
mknk1_ex14_rev	TAAAAGCGATGCGAATGTCA		
junba_fwd	TCACACCTGCAGCCAACTAC	Jun a proto-oncogene	400bp
junba_rev	TGAGCTACCTGCTCCCTCAG		
epha2_ex3_fwd	TGGTGAACGGCTCTCTTCTT	Ephrin Type-A Receptor 2 (transcript 1)	403bp

epha2_ex3_rev	GCCACACAGCCTCCTAAATC		
cyp24a1_fwd	AAAAGTCAACGGCAAAATGG	Cytochrome P450, family 24, subfamily A, polypeptide 1	384bp
cyp24a1_rev	TCTGTTGTGGCAGCGTAAAG		
gadd45bb_fwd	CTGCACAGCGACAAAACTGT	Growth arrest and DNA-damage-inducible, beta b	372bp
gadd45bb_rev	TAGAAGGCACCCACTGGTTC		
socs3b_fwd	CTCGGACCATCACCACTTCT	Suppressor of cytokine signaling 3b	396bp
socs3b_rev	ATGGGAGCATCGTACTCCTG		
hmox1a_fwd	GGAAGAGCTGGACAGAAACG	Heme oxygenase (decycling) 1a	405bp
hmox1a_rev	TCCTGCCTCTTTTGCTCTGT		
hsp70l_fwd	GGCACCACCTACTCCTGTGT	Heat shock cognate 70-kd protein, like	386bp
hsp70l_rev	GTCACCTTCTGCCCCAGATA		
mmp-9_ex4_fwd	CTCACGCTTATCCTCCAGGT	Matrix metalloproteinase 9	402bp
mmp-9_ex6_rev	TTGAAAAACAAAATGGTGAAGAAA		
foxo1a_fwd	GCAGAATGAGGGGACTGGTA	Forkhead box O1 a	390bp
foxo1a_rev	GAGGTCATTTTGGTCCCTGA		
il6st_fwd	CACATCATTGGCTGTTGGTC	Interleukin 6 signal transducer	383bp
il6sf_rev	TGGTTTCATCGGAGGGTATC		
vsig-10_fwd	AACCCGTCAGTGCATCTTTC	V-set and immunoglobulin domain containing 10	384bp
vsid-10_rev	CCCTTGATCTAGTGGCTGGA		
iitga11a_fwd	CCAACAACACATTGCTGGAG	Integrin, alpha 11a	394bp
iitga11a_rev	TGAACCGTCCAAGACAATCA		
Itgb4_fwd	ATTGCTGAGCTGGGACAGTT	Integrin, beta 4	416bp
Itgb4_rev	TTCCCACAGACCAGATCTCC		
**Down-regulated genes**			
		**Zebrafish (RT-PCR)**	
**primer ID**	**primer sequence**	**gene name**	**product length**
cnot7_ex3_fwd	GTAGCGAGACCAATCGGAGA	CCR4-NOT transcription complex, subunit 7	400bp
cnot7_ex5_rev	CGACCTCTTCATCTGGCAAC		
elf3_ex3_fwd	AAACCTGAGCGGCTATTCTTC	E74-like factor 3 (ets-domain TF, epithelia-specific)	402bp
elf3_ex4_fwd	TGGAGGGAAATGAGTTCTGC		

klf4a_ex2_fwd	TTCTCCATCAGGAGGTTTCG	Kruppel-like factor 4	404bp
klf4a_ex2_rev	CCTGCGGAAATCCAGAATAA		
mc1r_fwd	TGGCAATCATCAAGAACAGG	Melanocortin 1 receptor	401bp
mc1r_rev	AGTCACGAGACAGGCGATG		
npas4a_ex7_fwd	GAAGCCTCACCTGGAAAACA	Neuronal PAS domain 4	401bp
npas4a_ex7_rev	TCAGGAGTGGAGGGAGAAGA		
itpkb_ex2_fwd	GTTCAACATTCAGCCCACCT	Inositol-trisphosphate 3-kinase B	400bp
itpkb_ex2_rev	GTAAGTTGGACAGGGGCAAA		
cngb3_ex6_fwd	CTCCACCTCAACGCTTGTCT	Cyclic nucleotide gated channel beta 3	390bp
cngb3_ex8_rev	CAACATGCCCTGGGAGTC		
tnfb_ex2_fwd	GAAGGTCAGAAACCCAACAGA	TNF superfamily, member 2	401bp
tnfb_ex2_rev	CAAAACCCAATGTTGGATCA		
nfkbiaa_ex1_fwd	TCTCTCGGGCTTCACTTGTT	Nuclear factor of kappa light polypeptide gene enhancer in B-cells inhibitor, alpha a	396bp
nfkbiaa_ex2_rev	GTCGTTCTGGCACTGTTTGA		
ftr53_ex4_fwd	ATCAGCACCCCTTGAAATTG	FinTRIM family, member 53	401bp
ftr53_ex6_rev	ACACACCATAAGGCCCACTC		
egr_ex2_fwd	ACCTACACCGGCAGCTTCTA	Early growth response 4	396bp
egr_ex2_rev	CTGCTTGATCCAAGTGTCCA		

Table S5. Uncharacterized differentially regulated protein-coding transcripts and lincRNA (H_2O_2 vs. untreated zebrafish larvae)

Gene	Homologous to:	Chr	Begin	End	logFC	*P*	Ensembl Gene ID
BX005175.1	IKNASE C TYPE EC_2.7.11.13 NPKC	11	18765417	18789668	0.49	0.02	ENSDARG00000070651
BX248582.1	UNKNOWN	15	663267	668300	-0.65	0.04	ENSDARG00000009402
BX323045.3	CTD SMALL PHOSPHATASE	11	5851978	5870846	0.49	0.03	ENSDARG00000089646
BX323819.1	ADENYLATE CYCLASE TYPE EC_4.6.1.1	17	33519383	33544148	-0.64	0.03	ENSDARG00000043141
BX548011.3	UNKNOWN	4	49787861	49789795	1.30	0.01	ENSDARG00000087506
BX664721.5	UNKNOWN	7	76692360	76699575	-0.53	0.03	ENSDARG00000090371
BX908793.1	RHO GTPASE ACTIVATING	3	22599643	22634992	-0.57	0.01	ENSDARG00000061798
BX927362.1	SYNTHASE	6	43369376	43380209	0.98	0.00	ENSDARG00000087873
BX927369.2	UNKNOWN	17	49675395	49685460	-0.82	0.01	ENSDARG00000070652
BX957278.1	UNKNOWN	7	20975567	20983342	1.51	0.00	ENSDARG00000010940
CABZ01007794.1	UNKNOWN	7	2637136	2654723	1.03	0.00	ENSDARG00000088655
CABZ01020840.1	UNKNOWN	4	4474961	4485224	0.82	0.01	ENSDARG00000090401
CABZ01024770.1	ARF GAP WITH RHO GAP DOMAIN ANK REPEAT AND PH DOMAIN CONTAINING CENTAURIN DELTA CNT	21	42585816	42628903	0.64	0.01	ENSDARG00000087492
CABZ01041493.1	UNKNOWN	18	45219884	45238864	0.46	0.02	ENSDARG00000088409
CABZ01041812.1	FERRIC CHELATE REDUCTASE STROMAL CELL DERIVED RECEPTOR 2 SDR 2	24	30741886	30760340	1.51	0.00	ENSDARG00000061481
CABZ01067072.1	IMMUNOGLOBULIN SUPERFAMILY MEMBER 3 PRECURSOR IGSF3	9	520131	534160	0.68	0.02	ENSDARG00000086721
CABZ01072534.1	UNKNOWN	1	41080395	41120369	-0.64	0.03	ENSDARG00000075951
CABZ01076094.1	COLLAGEN ALPHA 1 IX CHAIN	19	4505333	4541542	0.59	0.04	ENSDARG00000039462
CABZ01076801.1	KINESIN	20	47775841	47801501	-0.43	0.04	ENSDARG00000017162
CABZ01080370.1	POU DOMAIN CLASS 2 TRANSCRIPTION FACTOR OCTAMER	1	628783	640887	0.74	0.00	ENSDARG00000043682
CABZ01084007.1	CENTROSOMAL OF 120 KDA CEP120 COILED COIL DOMAIN CONTAINING 100	8	88184	136657	0.46	0.03	ENSDARG00000091326
CABZ01084952.1	UNKNOWN	2	73298	80311	0.62	0.05	ENSDARG00000086333
CABZ01087111.1	MIDNOLIN MIDBRAIN NUCLEOLAR	16	52537808	52544134	1.55	0.00	ENSDARG00000087923
CR391977.3	UNKNOWN	25	27621529	27624918	-0.44	0.04	ENSDARG00000090185
CR391986.1	UNKNOWN	17	24745510	24765397	0.96	0.00	ENSDARG00000078130
CR391991.1	PERIOD CIRCADIAN HOMOLOG CIRCADIAN	2	48911934	48912326	1.61	0.00	ENSDARG00000078099
CT027835.1	ARYL HYDROCARBON INTERACTING	14	42351111	42369582	-0.67	0.02	ENSDARG00000014095
CU074314.1	RHO GUANINE NUCLEOTIDE EXCHANGE FACTOR 37	14	25848223	25872579	0.56	0.02	ENSDARG00000002463
CU459072.2	UNKNOWN	17	50819580	50836315	0.74	0.02	ENSDARG00000087251
CU469311.1	LYSYL OXIDASE HOMOLOG PRECURSOR	10	20441709	20480325	0.84	0.01	ENSDARG00000091492
CU633999.1	COLLAGEN ALPHA 1 CHAIN	19	61882	82475	0.71	0.04	ENSDARG00000076623
CU638738.1	PROTO ONCOGENE C FOS	17	50755160	50764033	-0.84	0.00	ENSDARG00000040135
CU638740.1	RETINOL DEHYDROGENASE	14	4329760	4340102	0.53	0.02	ENSDARG00000074971
CU915778.1	VON WILLEBRAND FACTOR A DOMAIN CONTAINING 7 PRECURSOR	1	44449883	44467566	-0.68	0.04	ENSDARG00000086717
FP236810.2	TYROSINE PHOSPHATASE AUXILIN EC_3.1.3.48 DNAJ HOMOLOG SUBFAMILY C MEMBER 6	5	9812700	9823111	0.49	0.05	ENSDARG00000090654
si:ch211-132b12.7	UNKNOWN	18	38965706	38981883	0.88	0.01	ENSDARG00000068374
si:ch211-161h7.5	UNKNOWN	24	21518374	21523502	0.94	0.01	ENSDARG00000093044
si:ch211-225b11.1	SODIUM AND CHLORIDE DEPENDENT TRANSPORTER SOLUTE CARRIER FAMILY 6	5	22570100	22597916	1.06	0.00	ENSDARG00000002405

	MEMBER						
si:ch211-241e1.3	LAMININ SUBUNIT ALPHA 3 PRECURSOR	2	1128112	1169253	1.47	0.00	ENSDARG00000022615
si:ch73-25i22.2	UNKNOWN	18	5109404	5124138	1.16	0.00	ENSDARG00000088549
si:dkey-104n9.1	UNKNOWN	8	13360832	13365015	1.40	0.00	ENSDARG00000093042
si:dkey-193c22.1	UNKNOWN	5	41825948	41835577	-0.85	0.01	ENSDARG00000093687
si:dkey-22f5.9	UNKNOWN	21	29414118	29421574	-1.10	0.03	ENSDARG00000092044
si:dkey-242g16.2	UNKNOWN	10	18017268	18018999	-0.80	0.01	ENSDARG00000069377
TTC17	TETRATRICOPEPTIDE REPEAT 17	7	51752705	51806323	0.40	0.03	ENSDARG00000044812
wu:fj08f03	UNKNOWN	14	11649673	11655264	0.62	0.01	ENSDARG00000077169
zgc:101549	SIALIC ACID SYNTHASE	25	5627495	5639284	-0.71	0.01	ENSDARG00000036009
zgc:103625	UNKNOWN	3	47690300	47695875	-0.57	0.03	ENSDARG00000038770
zgc:109934	FERRITIN HEAVY CHAIN	3	32255646	32257330	1.26	0.00	ENSDARG00000094210
zgc:112160	CHYMOTRYPSINOGEN B PRECURSOR	7	36516809	36521137	-0.83	0.01	ENSDARG00000039730
zgc:113054	UNKNOWN	6	43381521	43406057	0.70	0.02	ENSDARG00000053483
zgc:158706	UNKNOWN	21	9672503	9675872	1.37	0.00	ENSDARG00000069988
zgc:162184	CALPAIN SUBUNIT	17	45821870	45846739	1.22	0.00	ENSDARG00000077217
zgc:172139	UNKNOWN	4	60272405	60301556	0.77	0.01	ENSDARG00000068830
zgc:66382	TRYPSIN PRECURSOR EC_3.4.21.4	16	28253006	28254288	-1.08	0.01	ENSDARG00000079274
zgc:91849	IMMUNOGLOBULIN SUPERFAMILY MEMBER 2 PRECURSOR IGSF2	9	27602	39587	0.69	0.01	ENSDARG00000014323
si:dkeyp-86h10.3	Family: lincRNA	16	22398201	22399884	-0.76	0.04	ENSDART00000134980

Table S6. Top and Bottom 40-most statistically significant differentially regulated genes (H_2O_2 vs. untreated) from the RNA-seq larval zebrafish analysis

Rank	Symbol	logFC (H_2O_2 vs Untreated)	Rank	Symbol	logFC (H_2O_2 vs Untreated)
		Up-regulated			Down-regulated
1	hsp70l	2.66	1	npas4a	-2.23
2	mcm5	2.59	2	plekhf1	-1.84
3	mmp9	2.30	3	serpinh1b	-1.67
4	mmp13a	1.98	4	EGR4	-1.55
5	cyp24a1	1.84	5	nfkbiaa	-1.44
6	fosl1a	1.79	6	lrata	-1.28
7	cyp1a	1.76	7	fabp11b	-1.24
8	cry5	1.70	8	GPR3	-1.20
9	plek2	1.59	9	noxo1a	-1.19
10	per2	1.43	10	elf3	-1.18
11	wdr76	1.43	11	kcnj1a.5	-1.17
12	cbx7a	1.35	12	cnot7	-1.13
13	cry-dash	1.33	13	kcnj1a.3	-1.12
14	igfbp1a	1.29	14	cyr61	-1.11
15	sqstm1	1.25	15	CHORDC1	-1.10
16	ctssb.1	1.20	16	kcnj1a.4	-1.05
17	dnajb1b	1.20	17	guca1c	-1.03
18	cebpb	1.16	18	cry2a	-1.01
19	itgb4	1.15	19	ca4b	-1.01
20	bach1	1.13	20	dnaja1l	-0.97
21	junbb	1.10	21	grk1b	-0.97
22	abhd4	1.10	22	aldh3b2	-0.96
23	hsd17b12a	1.09	23	jund	-0.95
24	APOLD1	1.08	24	CNGB3	-0.95
25	bcl6ab	1.06	25	RGS9BP	-0.95
26	keap1a	1.06	26	muc13a	-0.94
27	junba	1.06	27	IMPG2	-0.94
28	hebp2	1.05	28	CINP	-0.91
29	pck1	1.04	29	ace2	-0.90
30	abcb6a	1.03	30	obfc1	-0.86
31	grhl3	1.02	31	kcnj1a.1	-0.84
32	itga6b	1.00	32	glmnb	-0.82
33	scd	0.98	33	znf532	-0.82
34	vtg7	0.94	34	mettl7a	-0.81
35	egln3	0.92	35	eif3bb	-0.81
36	lsm10	0.91	36	impg1a	-0.79
37	irg1l	0.89	37	ugt5d1	-0.78
38	AP1M2	0.89	38	hsp90aa1.2	-0.78
39	LDLR	0.87	39	kcnj1a.6	-0.78
40	lonrf1	0.86	40	rbm4.1	-0.77

FDR≤0.1

Table S7. Validation of RNAseq vs. qPCR

Gene	logFC RNAseq	logFC qPCR
eph2	0.67	0.55
junba	1.11	0.83
mknk1	0.68	0.37
odc1	0.72	0.25
plek2	1.55	2.60
sqstm1	1.32	0.66
junbb	1.19	1.19
xpc	0.71	0.64
cyp1a	1.76	2.99
scd	0.96	1.04
mmp13a	2.01	1.53
apold1	1.17	1.83
junba	1.11	0.74
elf3	-1.07	-0.94
klf4a	-0.94	-2.00
cnot7	-1.23	-1.24
mc1r	-1.13	-0.80
npas4	-2.21	-2.97
itpkb	-0.63	-0.30
fosl1	1.79	2.71
egfr	1.01	0.14
cngb3	-0.88	-1.05
tnfb	-2.86	-1.30
nfkbiaa	-1.34	-0.73
ftr53	-1.41	-0.62
egr4	-1.47	-1.80
cyp24a1	1.80	1.30
gadd45bb	0.80	0.60
socs3b	0.70	1.00
hmox1a	0.90	1.00
hsp70l	2.50	1.50
itgb4	1.20	0.70
vsig10	0.67	0.54
itga11a	0.37	0.71
foxo1a	0.46	1.08
il6st	0.53	0.54
mmp9	2.20	1.80
tgfb1b	0.30	0.90
g6pca.1	0.50	1.20
g6pca.2	0.46	0.90

Table S8. Correlation of 41 overlapping genes (GSE46343 vs. RNA-seq)

Genes	GSE46343 (logFC)	RNA-seq (logFC)
**ABCD4**	**-2.1**	**-0.5**
ALDH3B2	1.3	-1.0
**ATF3**	**2.0**	**0.7**
**ATF6B**	**1.2**	**0.4**
**ATG16L2**	**-1.1**	**-1.7**
CAV2	1.3	-0.5
CHD3	1.3	-0.6
CPT1B	-1.2	0.6
**CYP24A1**	**1.4**	**1.8**
EDC4	-1.3	0.5
**EGFR**	**1.4**	**1.1**
**ENC1**	**1**	**0.4**
**ERCC6**	**1.1**	**1**
**FASN**	**1.7**	**0.5**
FHOD1	-1.8	0.5
**FOSL2**	**2.4**	**0.4**
**FRRS1**	**2.6**	**1.5**
GRHL3	-1.2	0.4
**HMOX1**	**1.1**	**0.8**
**HSPA1l**	**1**	**2.7**
**IL6ST**	**2.5**	**0.5**
LIPG	-1.5	0.4
LSM10	-1.1	0.9
**MAFF**	**1.2**	**0.4**
MKNK1	-1.1	0.6
**MMP13**	**1.1**	**2.4**
**NANS**	**-1.3**	**-0.3**
NISCH	-1.1	0.5
NRF1	-1.2	0.7
**NUDT1**	**-1.2**	**-0.8**
**NUDT13**	**-1.5**	**-0.9**
**PIH1D1**	**-1.2**	**-0.7**
PIR	-1.0	0.4
**RALGPS2**	**2.2**	**0.8**
SLC23A2	-1.1	0.7
SORBS3	1.8	-0.5
TSPO	-1.4	0.4
**VSIG10**	**1.2**	**0.7**
XPC	-1.5	0.6
ZNF574	-1.4	0.8
**ZNF692**	**-1.5**	**-1.8**

note: bolded are correlated/congruent genes used for functional clustering analysis
